# Serrano (Sano) Functions with the Planar Cell Polarity Genes to Control Tracheal Tube Length

**DOI:** 10.1371/journal.pgen.1000746

**Published:** 2009-11-26

**Authors:** SeYeon Chung, Melissa S. Vining, Pamela L. Bradley, Chih-Chiang Chan, Keith A. Wharton, Deborah J. Andrew

**Affiliations:** 1 Department of Cell Biology, Johns Hopkins University School of Medicine, Baltimore, Maryland, United States of America; 2 Departments of Pathology and Molecular Biology, University of Texas Southwestern Medical Center, Dallas, Texas, United States of America; Harvard Medical School, Howard Hughes Medical Institute, United States of America

## Abstract

Epithelial tubes are the functional units of many organs, and proper tube geometry is crucial for organ function. Here, we characterize *serrano* (*sano*), a novel cytoplasmic protein that is apically enriched in several tube-forming epithelia in *Drosophila*, including the tracheal system. Loss of *sano* results in elongated tracheae, whereas Sano overexpression causes shortened tracheae with reduced apical boundaries. Sano overexpression during larval and pupal stages causes planar cell polarity (PCP) defects in several adult tissues. In Sano-overexpressing pupal wing cells, core PCP proteins are mislocalized and prehairs are misoriented; *sano* loss or overexpression in the eye disrupts ommatidial polarity and rotation. Importantly, Sano binds the PCP regulator Dishevelled (Dsh), and loss or ectopic expression of many known PCP proteins in the trachea gives rise to similar defects observed with loss or gain of *sano*, revealing a previously unrecognized role for PCP pathway components in tube size control.

## Author Summary

Tubular organ formation is a ubiquitous process required to sustain life in multicellular organisms. In this study, we focused on the tracheal system of the fruit fly, *Drosophila melanogaster*, and identified Serrano (Sano) as a novel protein expressed in several embryonic tubular organs, including trachea. *sano* loss results in over-elongated trachea, whereas Sano overexpression causes shortened trachea, suggesting that *sano* is required for proper tracheal tube length. Interestingly, Sano overexpression results in typical planar cell polarity (PCP) defects in many adult tissues and pupal wing cells. The PCP pathway is highly conserved from flies to mammals and it has been known to control cell polarity within the plane of epithelial tissues. Importantly, we found that Sano binds Dishevelled (Dsh), a key PCP regulator, and loss or ectopic expression of many known PCP proteins in the trachea give rise to similar defects observed with loss or gain of *sano*, suggesting a new role for the PCP genes in tube length control. Interestingly, the changes in tube length and PCP defects in the wing were linked to changes in apical domain size, suggesting that Sano and the PCP components affect either membrane recycling and/or the linkage of the membrane to the cytoskeleton.

## Introduction

Multicellular animals employ tubular structures in organs to transport vital fluids and gases that sustain life. Examples of organs with prominent tubular architecture include the circulatory system, the lung and kidney in mammals, the secretory and respiratory organs in flies, and the excretory organ in worms. Proper development of tubular networks is critical for the function of several organs, evidenced by disruption of these networks being an underlying cause of common human diseases including cardiovascular disease, polycystic kidney diseases, and asthma.

The *Drosophila* trachea is a branched network of tubular epithelia that transports oxygen and other gases throughout tissues. The comparative simplicity and genetic tractability of this system has made it one of the most powerful model systems to dissect tubular epithelial morphogenesis. Tracheal formation begins as tracheal placodes invaginate from the epidermis during early embryogenesis. Through stereotypic cell migrations, cell shape changes, and rearrangements of cell-cell junctions, tracheal cells generate a tubular network that extends branches to all embryonic tissues [1]–[4].

Each tracheal branch assumes a specific size as a consequence of branch-specific signaling events [5]–[10]. Tube size control is mediated by changes in cell shape, cell arrangement, and possibly cell size, but does not involve changes in cell number [11]. One category of genes that affect tube size encodes components of septate junctions, as mutations cause overelongated trachea [12]–[17]. Defects in apical extracellular matrix (ECM) proteins - which modify the structure of the chitin matrix - also lead to overelongated trachea, indicating that a dynamic and highly patterned apical extracellular matrix (ECM) regulates epithelial cell shape and tube size [18]–[22].

In epithelia, cells are polarized along the apical/basal axis. In epithelial tubes, the apical surface of each cell faces the lumen, whereas the basal surface faces surrounding tissues and/or a basement membrane. In addition to apical/basal polarity, epithelial cells in most tissues require information about their orientation within the plane, orthogonal to the axis of apical/basal polarity, in order to generate polarized structures such as cilia, or to move or orient themselves in a directed fashion. This type of polarization is referred to as planar cell polarity (PCP). In vertebrates, PCP is involved in diverse patterning events, including convergence extension during gastrulation, neural tube closure, inner ear sensory hair morphogenesis, and hair follicle orientation [23]. In *Drosophila*, PCP biases cell orientation in several adult epithelial tissues and has been implicated in ovarian border cell migration [24]–[27]. In many contexts, both in vertebrates and in *Drosophila*, a conserved PCP pathway – the Frizzled (Fz) pathway - mediates local cell-cell interactions that instruct neighboring cells to adopt appropriate polarity [24]–[27].

In *Drosophila*, loss or overexpression of PCP proteins causes disorganization of wing hairs and bristles on the thorax and/or alteration in the orientation of ommatidia in the compound eye. Analysis of such phenotypes revealed an evolutionarily conserved set of genes that control planar polarity – the “core” PCP factors. These factors include: Fz, a seven-pass transmembrane receptor [28]; Dishevelled (Dsh), an adaptor protein that acts downstream of Fz [29]–[31]; Flamingo/Starry night (Fmi/Stan), a cadherin-family member with a seven-pass transmembrane domain [32],[33]; Strabismus/Vang Gogh (Stbm/Vang), a four-pass transmembrane protein [34],[35]; and Prickle (Pk) and Diego (Dgo), each cytoplasmic proteins that are associated with the apical membrane during PCP signaling [36],[37].

PCP pathway activity itself leads to polarized enrichment and distribution of core components in all *Drosophila* tissues analyzed to date. In pupal wing cells, core PCP proteins localize apico-laterally, partially overlapping with cellular junctions [17],[38], and each protein is enriched in a distal and/or proximal location in the cells during prehair formation [33], [37], [39]–[42]. The function of each core PCP protein is essential for the asymmetric accumulation of the other proteins.

The PCP signal from Fz/Dsh directs asymmetric cytoskeletal reorganization and polarized cell morphology, in part by activating RhoA/Rho1 [43] and its downstream effector, *Drosophila* Rho-associated kinase, Drok [44]. In the wing, RhoA signals via Drok, which regulates myosin II activity via phosphorylation of Spaghetti squash (Sqh), a *Drosophila* homolog of nonmuscle myosin II regulatory light chain (MRLC) [44],[45]. Additional PCP regulators include Fat (Ft) and Dachsous (Ds), two protocadherins that can interact in a heterophilic fashion across cell boundaries [46],[47], and the Golgi kinase Four jointed (Fj) [48],[49]. Fj and Ds are expressed in a gradient in the eye and wing, making these proteins attractive candidates for providing upstream global polarity cues [46],[47]. Alternatively, the Ft/Ds group may function in parallel to the core PCP proteins [50].

Recently, a role for PCP genes in regulating tube length and diameter by orienting cell divisions was demonstrated in vertebrate renal and gut epithelia [51],[52], but whether the PCP components affect tube geometry in *Drosophila* remains unknown. Here we identify *serrano* (*sano*), a novel protein that affects tracheal tube length in *Drosophila*. *sano* mutant embryos have elongated tracheal dorsal trunks (DTs), whereas overexpression of Sano results in shortened DTs. Sano directly binds the core PCP component Dsh, and tracheal morphology and geometry are similarly affected by alterations in Sano activity and PCP signaling. Our results implicate for the first time the PCP mutants in *Drosophila* tubular morphogenesis.

## Results

### *sano* Encodes a Cytoplasmic Protein That Is Apically Enriched

**Figure 1:**
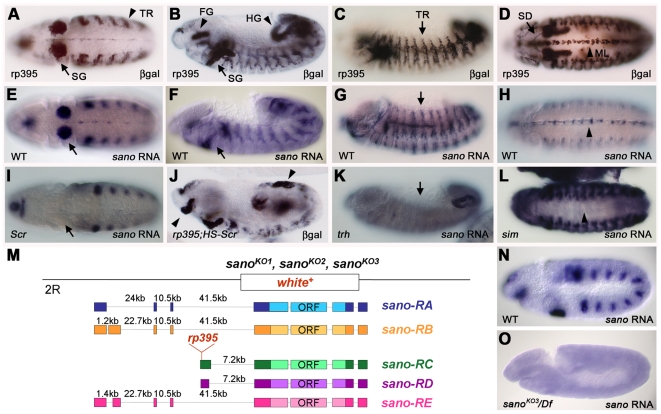
*sano* is dynamically expressed in many embryonic tissues. (A–D) *rp395* β-gal is expressed in the salivary gland (SG), salivary duct (SD), trachea (TR), CNS midline (ML), foregut (FG) and hindgut (HG). (E–H) *sano* RNA is expressed in the same pattern as *rp395* β-gal, except that expression is more transient. Note that expression in the SG (E) disappears by embryonic stage 13 (G, H). (I–L) *sano* is regulated by Scr (I, J), Trh (K), and Sim (L). Note the loss of *sano* in the salivary glands in the *Scr* mutant (arrow in I), the ectopic *rp395* expression in HS-Scr embryos (arrowheads in J), the loss of *sano* RNA in the trachea in the *trh* mutant (arrow in K) and the loss of *sano* RNA in the midline cells in the *sim* mutant (arrowhead in L). (M) There are five alternative *sano* splice forms, RA-RE, which encode the same ORF. The *rp395* P-element is inserted in the first exon of *sano-RC* and *sano-RD*. *sano^KO1^*, *sano^KO2^* and *sano^KO3^* are null alleles created by a targeted homologous recombination event in which the entire *sano* ORF was replaced with *white^+^*. (N, O) *sano* mRNA expression in st. 10 WT (N) and transheterozygous embryos of *sano^KO3^* over deficiency (O). *sano* patterns of expression were undetectable in *sano^KO3^/Df*.

An enhancer trap screen for lines with expression in the developing salivary gland and trachea identified *rp395*, a P-element insertion that expresses β-gal throughout the salivary gland, in trachea, and in several other embryonic tissues, including the hindgut, midgut endoderm, CNS midline, posterior spiracles, and epidermis (Figure 1A–1D). Cloning and characterization of the flanking region revealed that the *rp395* P element had inserted after nucleotide 14 of the *RC* and *RD* transcripts of *serrano* (*sano*; *CG12758*), two of five alternatively spliced transcripts, designated RA-RE (Figure 1M). Northern analysis revealed a single size transcript of 4.6 kb, first detected in 4–8-hour embryos and reaching peak levels in 8–12-hour embryos (Figure S1). The transcript was detected at all subsequent developmental stages, but was not detected in RNA isolated from cultured *Drosophila* Schneider (S2) cells. With minor exceptions, the endogenous *sano* transcripts recapitulate the pattern of *rp395* β-gal expression (Figure 1E–1H).

Sano expression requires the transcription factors Sex combs reduced (Scr) in the salivary gland, Trachealess (Trh) in the trachea, and Single-minded (Sim) in the CNS midline (Figure 1I–1L); *sano* expression was not affected by loss of transcription factors including *fork head*, *huckebein*, or *CrebA* that are expressed early in salivary gland formation (data not shown). Early transient tracheal expression of *sano* was observed in *trh* mutant cells also deficient for programmed cell death (*Df(3L)H99*), suggesting that initial tracheal expression is in part *trh*-independent and complete loss of *sano* expression in *trh* mutants is due to tracheal cell death (Figure S2). Since other known regulators of tracheal development, including *ventral veinless*/*drifter*, *trachea defective*/*apontic*, *breathless*, *branchless*, and *rhomboid*, did not affect *sano* expression (data not shown), initial *sano* expression could be regulated by factors that initiate *trh* expression. Since Trh and Sim bind the same consensus DNA sequence [53],[54], regulation of *sano* expression by these proteins could be direct.

**Figure 2:**
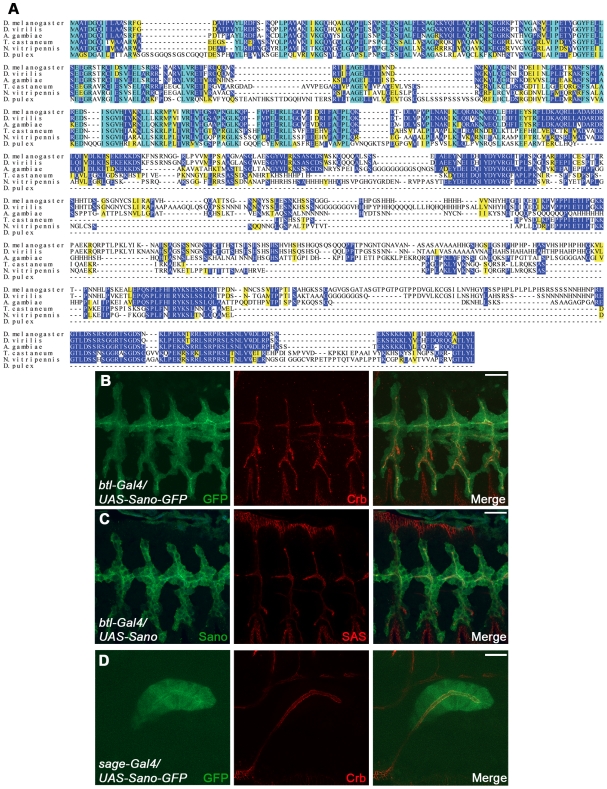
Sano protein is highly conserved in arthropods and localizes to the cytoplasm and apical membrane domain. (A) Sequence alignment of Sano with that of *D. virillis*, *A. gambiae*, *T. castaneum*, *N. vitripennis*, and *D. pulex*. Cyan, completely conserved residues in all species; Blue, identical residues; Yellow, similar residues. (B) GFP-tagged Sano protein was overexpressed in the trachea using *btl-Gal4*. GFP signal (green) is observed in the cytoplasm and is enriched in the apical domain where Crb, an apical membrane marker, localizes (red). (C) α-Sano antibody (green) recognized overexpressed Sano protein in the cytoplasm with apical enrichment in the trachea. Red, SAS. (D) GFP-tagged Sano protein was overexpressed in the salivary gland using *sage-Gal4*. Sano-GFP (green) was observed in the cytoplasm and was enriched in the apical domain where Crb localizes (red). Scale bars: 20 µm. Embryos in (B–D) are st.14.

**Figure 3:**
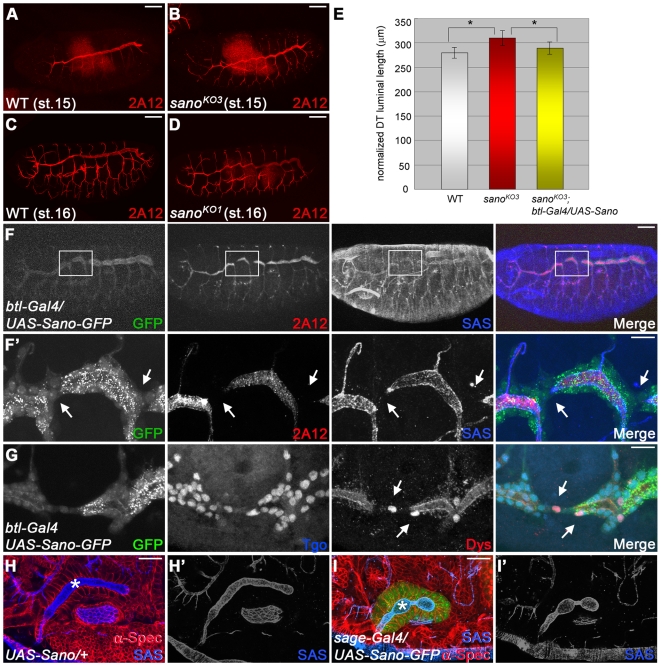
Sano affects tracheal tube length. (A–D) st.15 and st.16 WT (A, C) and *sano* mutant (B, D) embryos stained for 2A12. (E) Quantification of tracheal DT length in WT, *sano^KO3^* mutant and rescued embryos by *btl-Gal4*-driven overexpression of Sano in *sano^KO3^* mutant background. Error bars indicate standard deviation (SD) (*p<10^−3^, t-test). (F, F′) Overexpression of Sano results in shortened DTs (arrows) that fail to connect. Higher magnification of the boxed region is shown in F′. Green, *sano*-GFP; red, 2A12; blue, SAS. (G) A fusion marker is normally expressed in Sano-overexpressing trachea. Green, *sano*-GFP; blue, Tgo; red, Dys. (H, I) Sano overexpression causes shortened salivary gland lumen. Asterisks indicate salivary gland of WT (H) and *sage-Gal4>UAS-Sano-GFP* salivary gland (I). Scale bars: 50 µm in (A–D) and F, 10 µm for (F′, G), 20 µm for (H, I). Embryos in (F–H) are st.16.

All predicted *sano* splice forms encode the same 778-residue ORF (Figure 1M). Sano is highly conserved in arthropods (Figure 2A), and is a member of a largely uncharacterized family of proteins with members from cnidarians to mammals that includes the recently identified Themis protein (also known as Gasp). Themis/Gasp is a cytosolic thymocyte-adaptor protein that binds Grb2 and is required for positive selection of thymocytes [55]–[60]. Because we were unable to generate antiserum that detected endogenous Sano, we cloned and expressed both untagged and C-terminally tagged (GFP or HA) Sano in flies under Gal4/UAS control [61]. In both tracheal and salivary gland cells, each version of overexpressed Sano localized diffusely in the cytoplasm, with enrichment at apical membranes, colocalizing with the apical membrane markers Crumbs (Crb) and Stranded at Second (SAS) (Figure 2B–2D; [62]–[64]). During late embryogenesis and in the 3^rd^ instar larval salivary gland, however, Sano-GFP also localized to nuclei (Figure 3F, 3F′, and 3G and data not shown). Neither untagged (detected with Sano antiserum) nor HA-tagged Sano could be detected in nuclei at any stage. Taken altogether, these experiments suggest that Sano is an apically enriched cytoplasmic protein that may also sometimes localize to nuclei, a localization similar to that reported for the mammalian Themis/Gasp protein [55]–[60].

### *sano* Affects Tracheal Tube Length

Three independent loss-of-function knock-out *sano* alleles, *sano^KO1^*, *sano^KO2^* and *sano^KO3^*, were generated by homologous recombination [65]. PCR analysis confirmed that exons common to all five splice forms were replaced with the mini-*white^+^* gene (Figure S3A and S3B). *sano* mRNA was not detected in *sano* homozygotes or in embryos transheterozygous for each *sano* allele over a deficiency that removes *sano* and nearby genes, indicating that the *sano* alleles are null (Figure 1N and 1O; Figure S3C, S3D, S3E, S3F, S3G, and S3H; data not shown). Each *sano* allele is homozygous lethal, and lethal over the *sano* deficiency, with death occurring during the 2^nd^ instar larval stage. The *sano* lethality was partially rescued by expression of the Sano ORF under the control of a heat-shock promoter (HS-Sano) induced during larval stages (11/45 viable adults when heat shocked at 58–70 hr AEL).

Most features of salivary gland and tracheal development appear normal in *sano* mutant embryos (data not shown). Interestingly, however, staining with 2A12, a marker of tracheal lumen after stage 13, revealed that the dorsal trunk (DT) in *sano* mutants is more elongated and convoluted than in wild type (WT; Figure 3A–3D). Measurements of DT lengths from confocal projections of 2A12 staining from lateral views of stage 16 embryos revealed that *sano* mutant DTs are significantly (∼12%) longer than wild type (Figure 3E). Tracheal cell numbers in the dorsal trunk of *sano* mutants (14.7±0.6, N = 5, metamere 4) were comparable to those of WT (15.2±0.4, N = 5, metamere 4; p>0.5, t-test), indicating that the elongated DT phenotype is not due to increased cell numbers.

Conversely, Sano overexpression using *btl-Gal4* caused shortened DTs with discontinuous staining with either 2A12 or SAS (Figure 3F and 3F′). The UAS-Sano-GFP distributions in tracheal cells revealed that cells connecting adjacent segments of the DT (fusion cells) contact each other basally, but that the tracheal lumens and apical membranes are discontinuous. Fusion cell markers including Dysfusion (Dys), a bHLH-PAS transcription factor [66], and Arf-like-3 (Arl3), a small GTPase [67], were normally expressed in the discontinuous region of the DTs, indicating that fusion cells are not transformed to another fate (Figure 3G; data not shown). No increase in apoptosis was detected in the Sano-overexpressing trachea (Figure S5), and tracheal cell numbers in the *btl-Gal4:UAS-Sano* trachea (16±0.7, N = 7, metamere 4) were comparable to WT (15.2±0.4, N = 5, metamere 4; p>0.1, t-test), indicating that the shortened DT phenotype is not due to reduced numbers of tracheal cells. At 25°C, 100% of *btl-Gal4:UAS-Sano* embryos showed apical disconnection of DTs in more than one metamere, whereas neither *btl-Gal4* nor *UAS-Sano* alone had the shortened apical DT phenotype (Table 1). Sano overexpression also caused mismigration and/or failure of other tracheal branches to connect (data not shown). *btl-Gal4*-driven Sano expression in the trachea of *sano* null mutants rescued the elongated DT phenotypes observed in *sano* mutants (Figure 3E) and alleviated the gain-of-function phenotype of shortened DTs (Table 1), suggesting that an optimal dose of Sano is critical for proper tube length and that tube length is inversely related to Sano levels. Sano overexpression also reduced salivary gland lumenal length (88.7±2.0µm (WT) vs. 58.9±5.4µm (Sano-overexpressing glands), N = 5 for each genotype; p<0.01, t-test), suggesting that Sano has generalized effects on tube length (Figure 3H and 3I).

**Table 1:** Overexpression of Sano in the trachea causes shortened DTs.

	stage	shortened DTs (%)	normal DTs (%)	N
*btl-Gal4/UAS-Sano*	15	100	0	127
	16	98.4	1.6	63
*btl-Gal4/+*	15	1.8	98.2	109
	16	0	100	146
*UAS-Sano/+*	15	0	100	121
	16	0	100	162
*sano^KO3^*; *btl-Gal4/UAS-Sano*	15	60.8	39.2	130
	16	71.1	28.9	83

*All crosses were done at 25°C.

Tracheal tube size is controlled neither by the number nor the overall size of the individual cells [11]. Nonetheless, mutations in several genes have been discovered that, like loss of *sano*, lead to tracheal tube overelongation. Most of these known genes either regulate chitin synthesis or encode components of the septate junction, an invertebrate structure that has trans-epithelial barrier functions analogous to the vertebrate tight junction [16], [18]–[22],[68]. To test whether *sano* function is linked to either category of known genes affecting tube length, we analyzed luminal chitin using a fluorescent chitin binding protein (CBP) and a fluorescent chitin binding lectin (Wheat Germ Agglutinin; WGA) [21]. Both reagents revealed that the chitin cable, an extracellular scaffold upon which the tracheal branches elongate, is normal in *sano* mutants (Figure S4A and S4B; data not shown). *vermiform* (*verm*) encodes an apically-secreted chitin-binding protein with predicted polysaccharide deacetylase activity [12],[18],[22]. Verm staining in *sano* mutant trachea was indistinguishable from WT (Figure S4C and S4D). We conclude that tracheal length defects in *sano* mutants are not a consequence of detectable alterations in chitin biogenesis.

Septate junction proteins, including Coracle (Cor), Neurexin IV (NrxIV), and Fasciclin 3 (Fas3), localized normally to the basolateral domain of *sano* mutant tracheal cells, suggesting that septate junctions are intact (Figure S4E and S4F; data not shown). A 10 kDa dextran dye exclusion assay indicated that barrier function of septate junctions is intact in *sano* mutants (Figure S4G, S4H, and S4I). Thus, neither septate junction function nor chitin cable assembly is disrupted in *sano* mutants, suggesting another mechanism for the elongated tracheal phenotype.

### Sano as a PCP Regulator

**Figure 4:**
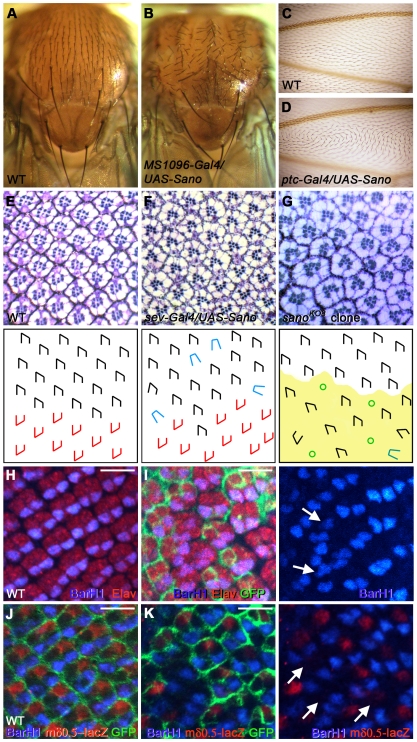
Overexpression of Sano causes PCP defects, and *sano* loss results in ommatidial defects. (A, B) Adult thorax of WT (A) and of *MS1096-Gal4>UAS-Sano* (B). Sano overexpression causes misorientation of thoracic bristles. (C, D) Adult wing of WT (C) and of *ptc-Gal4>UAS-Sano* (D). WT wing shows distal orientation of hairs, whereas overexpression of Sano causes a swirling hair pattern. (E–G) Adult ommatidia of WT (E), *sev-Gal4>UAS-Sano* (F) near the dorsal/ventral boundary, the equator, and *sano* mutant clones in the dorsal compartment (G). *sano* mutant cells have *w^+/+^* marker and they are distinguishable from the neighboring WT *w^−/−^* cells by the pigment around the cells. Schematic drawings are shown in the panels below the actual images with black and red shapes indicating the orientation of ommatidia normally found in the dorsal and ventral hemisphere of the eye, respectively, and blue shapes indicating a loss of ommatidial asymmetry. *sano* mutant cells are marked by the light green color in G and H. Green circles in H indicate ommatidia with abnormal photoreceptor number. (H, I) BarH1 expression in WT (H) and in *sano* mutant clones in the eye discs (I). BarH1 (blue), an R1 and R6 photoreceptor marker reveals ommatidial misrotation in the *sano* clones (arrows). Red, Elav. The absence of GFP signal indicates *sano^KO2^* mutant clones. Scale bar: 5 µm. (J, K) WT and *sano* mutant clones in the eye discs. mδ0.5-lacZ (red), a R4 photoreceptor marker is absent or significantly reduced in the *sano* clones (arrows). Blue, BarH1. The absence of GFP signal indicates *sano^KO2^* mutant clones. Scale bar: 5 µm.

**Figure 5:**
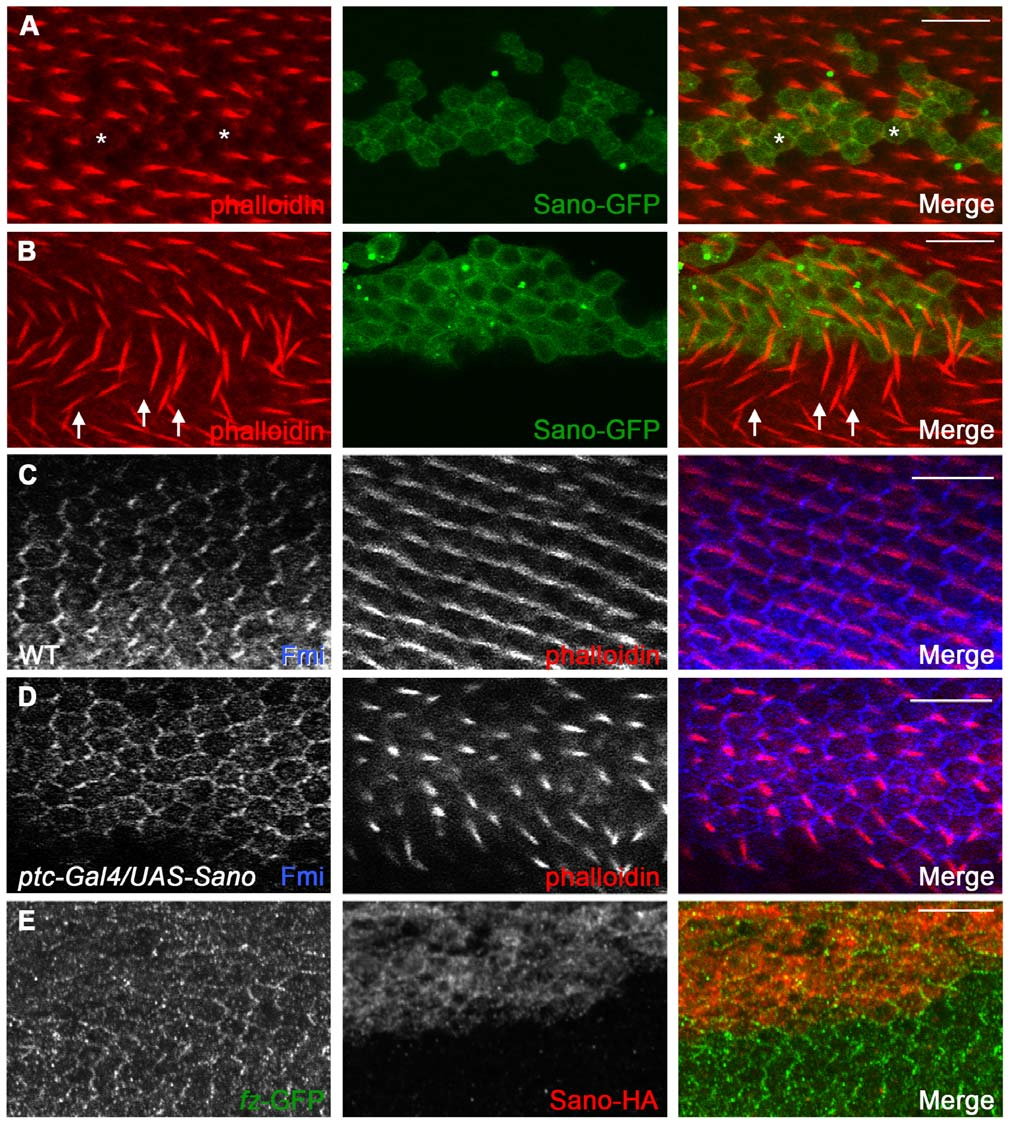
Overexpression of Sano causes PCP defects. (A) Prehair formation is delayed in Sano-overexpressing cells in the pupal wing (asterisks). (B) Sano overexpression causes nonautonomous misorientation of prehairs in specific regions of the pupal wing (arrows). (C, D) Fmi staining in WT and Sano-overexpressing pupal wing cells. Typical zigzag localization of Fmi (blue) and distal orientation of prehairs (actin, red) are shown in WT (C). In Sano-overexpressing cells, Fmi is observed all around the apical membrane and many hairs are misoriented (D). (E) *fz*-GFP (green) shows a fuzzy distribution throughout the apical margin in Sano-overexpressing clones (red). Scale bars: 10 µm.

*sano* is dynamically expressed in larval imaginal discs, structures that give rise to much of the adult during metamorphosis (Figure S6). Overexpression of Sano using several imaginal disc-specific Gal4 drivers caused planar polarity defects. For example, in the wild-type adult thorax, bristles point posteriorly, whereas in Sano-overexpressing adult thoraces, the bristles displayed altered orientations (Figure 4A and 4B). In the WT wing, each cell produces a single distally-oriented, actin-rich protrusion (a trichome, a.k.a. a “hair”). All Sano-overexpressing wing cells exhibited swirling hair patterns (Figure 4C and 4D). Sano overexpression in the eye caused ommatidial polarity defects, including misoriented and symmetrical photoreceptor phenotypes, as well as abnormal photoreceptor numbers (Figure 4E and 4F; data not shown), with about 14.5% of the ommatidia showing defects (137/916, N = 5). Polarity defects observed with Sano overexpression are similar to those observed when PCP genes are mutated or overexpressed [33], [36], [37], [43], [69]–[73], suggesting that Sano perturbs PCP. Next we examined Sano-overexpressing pupal wing cells. Phalloidin staining of actin-rich prehairs at 32 hours after puparium formation (APF) revealed that hair formation is delayed in Sano-overexpressing cells (Figure 5A), as observed in *dsh* mutant clones or in *dgo pk* double mutant clones [74]. Phalloidin staining of the slightly older pupal wings (at 33–34 hours APF) revealed Sano-overexpressing cells with prehairs the same size as surrounding wild-type hairs but with altered polarity (Figure 5B). Sano overexpression sometimes produced multiple wing hairs, another PCP phenotype (Figure S7B). Wild-type hairs near some sano-overexpressing clones exhibited non-cell-autonomous polarity defects (Figure 5B, arrows; Figure S7C and S7D), distinct from those near *fz* or *stbm*/*Vang* mutant clones; wild-type cells proximal to *fz* clones or distal to *stbm*/*Vang* clones have reversed hair polarity [34],[35],[75], which was not observed with Sano overexpression. All Sano overexpression clones that produced nonautonomous phenotypes mapped either between veins 3 and 4, distal to the anterior crossvein, or between veins 4 and 5, distal to the posterior crossvein (Figure S7A; N>100 clones examined), both regions of which are sensitive to PCP alteration [49].

Core PCP proteins are asymmetrically localized in pupal wing cells during prehair formation and show typical “zigzag” localization patterns on the apical surfaces of the pupal wings [33],[36],[37],[39],[40],[42]. When a PCP gene is mutated or overexpressed, other PCP proteins are typically mislocalized. Sano overexpression in pupal wings through either *ptc-Gal4*-driven expression or in Sano-overexpressing clones resulted in the mislocalization of all PCP proteins examined. Fmi, normally localized to both the proximal and distal sides of wing cells during prehair formation, was observed around the entire perimeter (Figure 5C and 5D). A similar mislocalization was observed with Stbm and Pk (Figure S8A; data not shown). Fz and Dsh, which normally localize to the distal side of the apical surface, exhibited reduced apical membrane distribution with Sano overexpression (Figure 5E, Figure S8B).

*sano* loss-of-function mutant clones in cells giving rise to adult tissues such as thorax and wing did not result in PCP phenotypes (data not shown). Similarly, actin prehairs of *sano* mutant clones in pupal wing cells always pointed distally as in WT (Figure S9A and S9B). Since mutations of some PCP genes, such as *ft*, show polarity defects only in very large clones [46], we induced *sano* mutant clones at earlier time points to generate a range of sizes of clones missing *sano* function. Even very large clones did not exhibit PCP defects (data not shown). However, although it was rare, when we induced clones relatively early (36–48 hours after egg laying (AEL), we obtained only twin spots (<5%, N = ∼70), suggesting that the *sano* mutant cells either died or were eliminated from the wing epithelium (Figure S9C and S9D). On the other hand, *sano* null eye clones had defects characteristic of loss of known PCP genes, including misoriented ommatidia and loss of asymmetry (Figure 4G; Table 2). *sano* null eye clones also often had abnormal numbers of photoreceptors (Figure 4G; Table 2). In 3^rd^ instar eye discs, the expression of BarH1, a marker for the R1 and R6 photoreceptors [76], showed ommatidial misrotation, consistent with the adult phenotype (Figure 4H and 4I), and the expression of *mδ0.5-lacZ*, a marker for the R4 photoreceptor, was absent or significantly reduced in *sano* null clones, consistent with a cell fate change of R4 to R3, which has been observed with some PCP mutants, including *fz* and *dsh* (Figure 4J and 4K; [77]). Our data suggest that although *sano* overexpression disrupts PCP signaling in multiple tissues, loss of *sano* results in a range of defects that are limited to fewer tissues.

**Table 2:** *sano* mutant eyes have various phenotypes including abnormal numbers of photoreceptors, loss of asymmetry, and misrotation of ommatidia.

abnormal photoreceptor numbers (%)	symmetrical ommatidia (%)	misrotation of ommatidia (%)	number of ommatidia scored	number of clones examined
24.0	5.8	20.7	208	6

### PCP Mutants Have Elongated DTs

**Figure 6:**
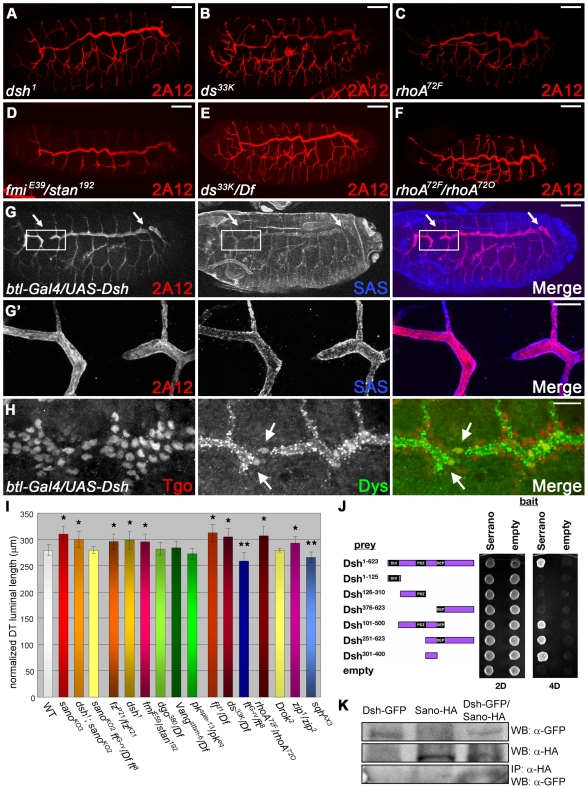
Many PCPm have tracheal length defects. (A–F) 2A12 staining in *dsh^1^* (A), *ds^33K^* (B), *rhoA^72F^* (C), *fmi^E59^/stan^192^* (D), *ds^33K^/Df(2L)Exel8003* (E), *rhoA^72F^/rhoA^72O^* (F) mutant embryos. (G, G′) *btl-Gal4*-driven overexpression of Dsh in the trachea results in a shortened DT phenotype similar to the Sano overexpression phenotype. Higher magnification of the boxed region is shown in G′. Red, 2A12; blue, SAS. (H) A nuclear fusion marker is normally expressed in Dsh-overexpressing trachea. Blue, Tgo; red, Dys. (I) Quantification of the length of DT of st. 16 PCP mutants. Error bars, SD (*, longer DT length, p<0.05, t-test; **, shorter DT length, p<0.05, t-test). (J) Yeast-two-hybrid assay using the full length and several fragments of the Dsh protein shows that the small fragment (∼100 a.a.) of Dsh between the PDZ and DEP domains binds to Sano. (K) co-IP experiment using embryo extracts confirms the interaction between Sano-HA and Dsh-GFP. Scale bars: 20 µm for (A–G), 10 µm for (G′, H). All embryos shown are st. 16.

To determine if *sano* affects tube length by altering PCP signaling, we asked if other PCP mutants have tracheal length defects, including null mutants of the core PCP genes *fz*, *dsh*, *fmi*, *dgo*, *stbm*, and *pk*, the *ft*/*ds* group of PCP regulator genes *fj*, *ft*, and *ds*, and the PCP downstream effectors *rhoA*, *Drok*, *zip* and *sqh*. For *dsh*, a key hub in canonical Wingless (Wg)/Wnt signaling and in Fz-dependent PCP signaling, we used the *dsh^1^* allele, which is defective for only its PCP function [69],[70]. Interestingly, many PCP mutants had tracheal length defects, exhibiting similar elongated DT phenotypes as loss of *sano* (Figure 6A–6F and 6I). Among the core PCP genes, *fz*, *dsh* and *fmi* had elongated DTs, whereas *dgo*, *pk* and *stbm* mutant embryos had normal DTs. Among the *ft*/*ds* PCP regulator group, *fj* and *ds* had elongated DTs. Among the PCP downstream effectors, *rhoA* and *zip* mutant embryos showed elongated DTs, revealing a potential role for the cytoskeleton in tracheal elongation. *Drok* mutant embryos also have convoluted trachea, but overall tracheal length was comparable to WT. *ft* and *sqh* mutant embryos had shorter DTs than WT, but the DTs were contiguous (Figure 6I). Overexpression of Dsh or a constitutively-active form of RhoA in the trachea caused shortened DT defects with discontinuities similar to *sano* overexpression (Figure 6G and 6G′; [78]), further implicating this pathway in apical cell surface elongation. Sano and Dsh are both cytoplasmic proteins, and Sano binds Dsh in yeast two-hybrid assays and co-immunoprecipitation (co-IP) (Figure 6J and 6K), providing a physical link between Sano and PCP proteins that is consistent with genetic interactions between *dsh* and *sano*; double mutants of *sano* and *dsh^1^* have elongation defects similar to those of *sano* or *dsh^1^* alone, suggesting that Sano and Dsh act in a common pathway (Figure 6I). Moreover, reduction of PCP function of Dsh (*dsh^1^/+*) suppressed the Sano overexpression phenotype in the thorax, a finding also consistent with Sano acting through Dsh (Figure S10). The apical enrichment of Dsh in the late embryonic trachea and Fmi localization to the adherens junctions (Figure S11) is consistent with PCP proteins acting at the apical membrane. These data suggest that Sano affects tube length by impinging on Dsh activity, likely through its role in PCP signaling. Also consistent with this model is our finding that *sano;ft* double mutant trachea have DT lengths that are intermediate between those of *ft* and *sano* mutants alone (Figure 6I).

### Sano Overexpression Results in Smaller Apical Domains

**Figure 7:**
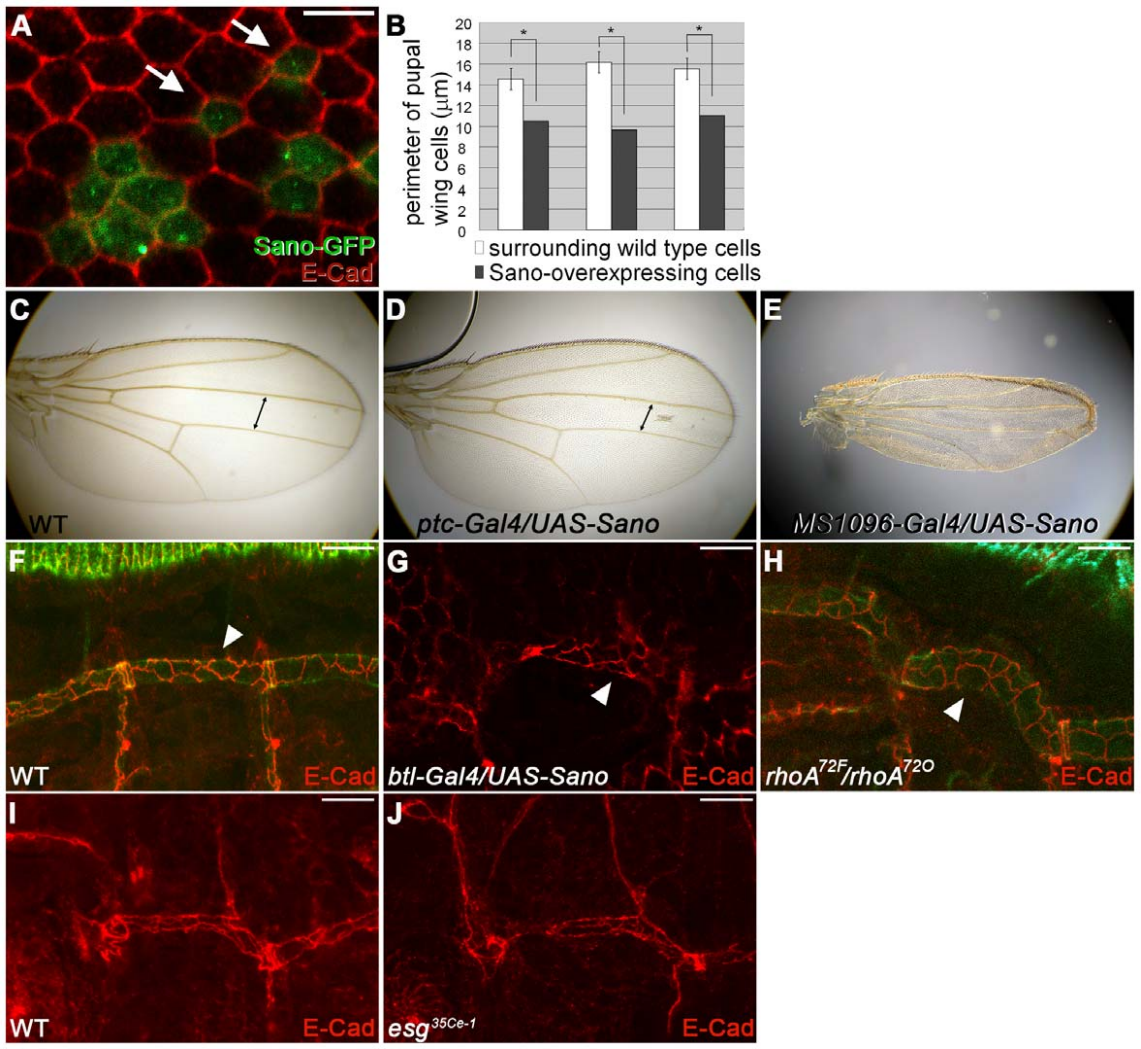
Overexpression of Sano results in smaller apical domains. (A) Sano-overexpressing pupal wing cells (green) are smaller than neighboring wild-type cells and often have a pentagonal rather than hexagonal shape (arrows). Red, E-Cad. (B) Quantification of the perimeter of three different clones of Sano-overexpressing single pupal wing cell and corresponding wild-type neighbors. Error bars, SD. *, p<0.05, t-test. (C–E) Adult wings. Sano overexpression at the anterior/posterior boundary with *ptc-Gal4* results in a narrower region between veins 3 and 4 (arrows in C and D). Sano overexpression in the entire wing with *MS1096-Gal4* results in a dramatic decrease in overall wing size. (F–H) E-Cad staining of tracheal cells (metamere 4). WT (F). *btl-Gal4*-driven Sano-overexpressing trachea (G) reveal a significant decrease in apical domain size. The tracheal cells in *rhoA* mutant embryos have larger apical domains (H). Arrowheads indicate apical domain of single cells in each allele. (I, J) E-Cad staining of tracheal cells (metamere 1). WT (I) and *esg* mutant trachea that have fusion defects (J) have comparable apical domain size. All of the embryos shown are st.16. Scale bars: 5 µm in (A), 1 µm in the inset, 10 µm in (F–J).

Since wing epithelial cells become hexagonally packed prior to PCP proteins regulating hair formation [79], we examined cell shape in Sano-overexpressing wing cells. As observed with other PCP mutants, Sano-overexpressing cells often assume a pentagonal shape instead of the typical hexagonal shape (Figure 7A; [79]). Sano overexpressing cells also have smaller apical domains than surrounding wild-type cells (Figure 7A; here, we define the apical domain as the area circumscribed by the zonula adherens, where E-Cad localizes). Using E-Cad staining, we measured the apical domain perimeters from several examples of single Sano-overexpressing cells and found a 29–41% decrease in the perimeters of the Sano-overexpressing cells compared to their wild-type neighbors (Figure 7A and 7B).

A decrease in apical domain size with Sano overexpression was also seen in adult tissues. For example, *ptc-Gal4*-driven Sano expression at the anterior-posterior wing margin resulted in a decreased distance between wing veins L3 and L4 compared to wild-type wings (Figure 7C and 7D; 178.29±2.10 pixels vs. 210.91±4.58 pixels, N = 3 for each genotype; p<0.005, t-test). Wing cell numbers in the area demarcated by veins L3, L4, the anterior crossvein, and an imaginary line starting from the tip of posterior crossvein and perpendicular to L3, did not reveal a significant difference in cell number between WT and *ptc-Gal4:UAS-Sano* wings (354.3±16.9 vs. 360±17.3, n = 3 for each genotype; p>0.5, t-test), indicating that the decrease of the adult wing size is due to a decrease in apical domain size. Likewise, global wing expression of Sano, using the *MS1096-Gal4* [80], resulted in a decrease in overall wing size (Figure 7E). To ask if *sano* affects tracheal tube length through changes in apical domain size, we examined E-Cadherin staining of WT, *sano* mutant, and Sano-overexpressing trachea. We also examined E-Cadherin staining in PCP mutants with altered tracheal tube length. Although it was difficult to ascertain differences in apical domain size of individual cells between WT and *sano* mutant trachea, which are expected to be at most ∼12% different, we observed a marked decrease in apical domain size in the Sano-overexpressing tracheal cells (Figure 7G). A similar decrease in apical domain size was observed in Dsh-overexpressing tracheal cells (data not shown). Moreover, the tracheal cells of *rhoA*, one of the PCP mutants, had larger apical domains than WT (Figure 7H), indicating that the changes in tube length observed with Sano and other PCP genes are due to altered cell geometry and not altered cell arrangement. In *escargot* (*esg*) mutant trachea, where infrequent DT breaks occur, apical domain size was comparable to WT, suggesting that the smaller apical domain size observed with *sano* and *dsh* overexpression is not a due to a failure of adjacent DT segments to fuse (Figure 7I and 7J).

## Discussion

Here, we report the discovery of Sano, a novel cytoplasmic protein enriched in the apical domains of developing tubular organs and other epithelia. Loss of *sano* results in overelongated tracheal tubes, whereas increased Sano shortens tracheal tubes, frequently leading to failures in dorsal trunk fusion. In adult tissues, Sano overexpression leads to characteristic PCP defects including misorientation of hairs on the adult wing, mislocalization of core PCP proteins in pupal wing cells, misorientation of thoracic bristles and loss of asymmetry in the rhabdomeres of the ommatidia. Based on clonal analysis, loss of *sano* does not cause PCP defects in the wing but is required for normal ommatidial organization. Further support for Sano functioning as a PCP regulator is provided by our finding that mutations in both global and core PCP genes, as well as in the PCP downstream effectors, result in tracheal length defects similar to those seen with loss of *sano*. Based on comparisons of Sano-overexpressing and wild-type cells, Sano limits apical membrane domain size, suggesting that Sano and known PCP regulators control the linkage of the subapical cytoskeleton to the apical membrane and/or modulate apical membrane trafficking by regulating levels of endocytosis and exocytosis.

### Identification of a Novel Protein Affecting PCP

Since the discovery that the Fz pathway controls PCP, many additional PCP components have been identified, including core factors, several PCP regulators, and general and tissue-specific downstream effectors [24],[25],[27]. Sano overexpression causes PCP defects in adult epithelial tissues as well as mislocalization of core PCP proteins. In wing cells, *sano* null cells appeared normal although we very occasionally obtained twin spot-only clones, suggesting a role for Sano in cell survival or in epithelial maintenance. It is unclear whether this function is related to PCP. On the other hand, *sano* loss in the eye gave rise to a range of defects, some of which are typical of PCP mutants, including loss of R4 cell specification, ommatidial misorientation, and loss of equatorial asymmetry.

The direct physical interaction between Dsh and Sano (Figure 6J and 6K) provides potential mechanistic insight into Sano function. The interaction between Dsh and Sano appears quite different from that between Dsh and Naked cuticle (Nkd), a Wingless (Wg) antagonist that also gives rise to PCP defects when overexpressed. Dsh participates both in canonical Wg/Wnt signaling and in Fz-dependent PCP signaling [29]–[31]. Like Sano, Nkd directly binds Dsh, and overproduced Nkd causes polarity defects and limits Wg signaling activity presumably by sequestering, degrading and/or modifying Dsh and thus blocking its participation in PCP [81]. Unlike Nkd, however, Sano overexpression does not cause defects typical of those seen when canonical Wg signaling is blocked. Moreover, whereas Nkd overexpression blocks Dsh activity, our studies of tube length control suggest that Dsh and Sano act in the same direction: gain or loss of Dsh mimics the gain or loss of Sano in the trachea. Similarly, *dsh sano* double mutants have the same tracheal length defects as each single mutant (Figure 6I). Likewise, overexpression of either Dsh or Sano in the eye using *sev-Gal4* causes similar changes in ommatidial polarity and rotation [82]). Finally, we showed that reduced *dsh* function suppresses the Sano overexpression PCP phenotypes in the thorax (Figure S10). Overall, the interaction and genetic data suggest that Sano and Dsh act together in a common pathway.

### A Role for PCP Signaling in Tube Size Control

It is intriguing that loss-of-function mutations in many, albeit not all, PCP genes result in similar tube elongation defects observed with loss of *sano* (Figure 6). PCP signaling can provide directional cues at the single cell level, such as directions on where to place the single hair within a *Drosophila* wing cell, or at the level of cell groups, such as controlling the organization of mechanosensory bristles in the *Drosophila* thorax and arrangement of photoreceptors in the *Drosophila* eye. PCP signaling also controls the behavior of cell populations undergoing extensive rearrangements, such as the dynamic morphogenetic changes that occur during body axis elongation in *Drosophila* and vertebrates and in ovarian border cell migration [23],[27],[83]. A recent study has implicated mammalian Fat4, a vertebrate homologue of the *Drosophila* global PCP protein Fat, in promoting renal tubule elongation through its effects on oriented cell divisions [51]. In those studies, loss of *Fat4* led to shorter renal tubules, a defect exacerbated by simultaneous loss of one copy of *Vangl2*, a vertebrate homologue of the core PCP protein Stbm/Vang. Consistent with this finding, our studies reveal that, in the trachea, mutations in the proteins that negatively regulate Fat (Ds and Fj) and the Stbm/Vang complex (Fz, Dsh) have the opposite defect: longer tubes. In the case of the trachea, a tissue whose final cell divisions occur much earlier in development than when Sano affects tube length, the effects of the PCP pathway are on cell shape rather than on the orientation of cell division. Whether the subcellular mechanisms by which PCP genes regulate oriented cell divisions in vertebrates and apical membrane elongation in flies are similar or distinct is not clear, but the parallels in the two systems provide evidence for evolutionarily conserved functions for PCP genes in tubular architecture.

The finding of a role for PCP genes in tube length control raises two crucial questions. (1) Are PCP proteins asymmetrically localized in tubular epithelia in the same way they are in wing, eye and border cells [23],[26],[27]? (2) How do PCP genes regulate tube length? We examined the subcellular localization of Dsh and Fmi in the tracheal cells, where Dsh localizes mainly in the cytoplasm and is enriched at the apical domain at later stages, and Fmi localizes to the adherens junctions (Figure S11). Unfortunately due to the irregular shape of tracheal cells and the three-dimensional structure of the tracheal tube, we could not determine with adequate resolution whether the PCP proteins are asymmetrically distributed. However, our data provides new insight into how PCP affects tracheal tube size. In the trachea, loss of *sano* or PCP function resulted in tubes that were 7–15% longer than WT based on apical domain measurements (2A12 staining). Since the *sano* trachea have the same number of cells as wild-type, each tracheal cell, on average, must have an apical domain that is approximately 12% longer than wild-type. Although an accurate measurement of apical dimensions in the trachea could not be obtained due to the shape and curvature of the tube, in *rhoA* mutant trachea, where the elongated DT defects were most obvious, the apical domains of the DT cells were consistently larger than WT (Figure 7H). Similarly, E-Cad staining in *dsh* and *ds* mutant tracheal cells revealed slightly larger apical domains (data not shown). Importantly, overexpression of Sano and Dsh resulted in tubes that were so much shorter than WT that the individual segments were often too short to anastomose. Examination of E-Cad staining in Sano- or Dsh-overexpressing trachea revealed markedly smaller apical domains in these cells, consistent with the decreased apical domain size observed in wing cells overexpressing Sano (Figure 7G; data not shown). Thus, PCP components appear to control overall tube length by limiting the size of the apical domain. This activity could be mediated by increased linkage of the plasma membrane to the underlying cytoskeleton and/or by direct effects on plasma membrane growth by modulating relative levels of exoctyosis and endocytosis. In support of a link between PCP signaling and regulated vesicle trafficking, Rab11/Sec5-dependent recycling of E-cadherin has been implicated in junctional remodeling during hexagonal packing of wing cells, wherein the polarized recruitment of Sec5 is through the PCP protein Fmi [79]. Importantly, tracheal tube elongation has also been linked to regulated vesicle trafficking through Rab11 [64],[84] and through core components of the secretory machinery [85],[86].

There are two potential inconsistencies with a model that Sano functions to control tube length through its effects on PCP signaling: (1) Loss of *sano* does not give rise to overt PCP defects in all adult tissues and (2) not all of the components of the PCP signaling pathway disrupt tube length when their function is missing. Indeed, loss of *ft*, which is expressed early in the tracheal primordia, appears to have effects opposite those of loss of *sano* on tube length. Although the tissue-specificity of *sano*'s role in PCP could reflect functional redundancy, it is also possible that *sano* and the large subset of the PCP genes that do have tracheal defects may function through novel, non-canonical, pathways to control tube length. In either case, it will be exciting to unravel the details of Sano's interactions with the cellular machinery to control apical domain size.

## Materials and Methods

### Flies and Antibodies

Fly strains used in this study were: *fz^P21^*, *fz^K21^*, *pk^sple-13^*, *MS1096-Gal4* (P. Adler); *Scr^4^*, *trh^1^*, *sim^2^*, *mega^EA97^*, *dsh^1^*, *stan^192^*, *stbm^6^*, *pk^eq^*, *rhoA^72F^*, *rhoA^72O^*, *Drok^2^*, *zip^1^*, *zip^2^*, *fj^d1^*, *ds^33K^*, *ft^Gv-5^*, *esg^35Ce-1^*, *Df(2L)Exel8003*, *Df(2R)ED2076*, *Df(2R)ED3610*, *Df(2R)Bsc271*, *sev-Gal4*, (Bloomington stock center); *Df(2R)Exel6068* (Exelixis); *btl-Gal4* (S. Hayashi); *sqh^AX3^* (L. Luo); *ptc-Gal4* (D. Pan); *HS-Scr* (M. Scott); *dgo^380^*, *mδ0.5-lacZ* (D. Strutt); *fmi^E59^* (T. Uemura); *sage-Gal4* (A.Vaishnavi and D.J.A., unpublished).

The primary antibodies used were mouse α-β-gal (Promega, 1∶500), rabbit α-GFP (Molecular Probes, 1∶10,000), mouse α-HA (Roche, 1∶500), mouse 2A12 (DSHB, 1∶10), mouse α-Crb (DSHB, 1∶10), rabbit α-SAS (D. Cavener, 1∶500), mouse α-α-Spec (DSHB, 1∶1), rat α-DE-Cad (DSHB, 1∶10), mouse α-Fmi (DSHB, 1∶10), rat α-Dsh (T. Uemura, 1∶1,000), rabbit α-Stbm (T. Wolff, 1∶200), rabbit α-Pk (J. Axelrod, 1∶2,000), guinea pig α-Verm (C. Samakovlis, 1∶500), CBP-FITC (New England BioLabs, 1∶500), WGA-488 (Molecular Probes, 1∶1000), guinea pig α-Cor (R. Fehon, 1∶2,000), rabbit α-NrxIV (H. Bellen, 1∶2,000), mouse α-Fas3 (DSHB, 1∶10), rabbit α-Dys (S. Crews, 1∶800), rabbit α-Arl3 (S. Hayashi, 1∶2,500), mouse α-Elav (DSHB, 1∶250), and rat α-BarH1 (H. McNeil, 1∶1000). Fluorescence-labeled secondary antibodies were used at a 1∶500 dilution (Molecular Probes).

### Immunohistochemistry

Embryo fixation and staining were performed as described [87] except for the α-E-Cad staining, for which embryos were fixed in 4% paraformaldehyde in PBS and devitellinized with ethanol. 3^rd^ instar *rp395* larval discs were dissected and fixed in 2% paraformaldehyde in PBS for 20 minutes, incubated with primary antibody overnight (4°C) and then with the appropriate secondary antibody for two hours (RT).

### Whole-Mount *in situ* Hybridization on Embryos and Imaginal Discs

*In situ* hybridizations were performed as described by [88]. The pPB3 cDNA, isolated by screening a cDNA library provided by L. Kauvar, was used to generate an anti-sense digoxigenin-labeled *sano* RNA probe.

### Identification of Sano

*sano* was identified in a P-element expression screen in Corey Goodman's laboratory. We obtained the *rp395* line because of its salivary gland and tracheal expression. Sano was independently identified in an EP screen for genes that when misexpressed alter the eye phenotype of Dsh+Nkd overexpression (S. Silva, G. Celik, C.-C. C. and K.A.W., unpublished).

### *sano* Null Alleles

Null *sano* mutants were generated by homologous recombination [65]. Genomic fragments upstream and downstream of the *sano* ORF were amplified by PCR and cloned into pW25, which carries *white^+^*, the recognition site for I-SceI endonuclease, and FRT sites. The construct was injected into embryos by Rainbow Transgenic Flies, Inc. Transformants were crossed to flies carrying hs-I-SceI and hs-Flp and progeny were heat shocked (37°C) for 1 hour 48–72 hours AEL.

### Transgenic Flies

The *sano* ORF was PCR-amplified and cloned into the pUAST [61] or pABAL expression vector [89] to create UAS-Sano and HS-Sano. UAS-Sano-GFP and UAS-Sano-HA were created using the *Drosophila* Gateway Vector system (Carnegie Institution).

### Sano Antibody

A PCR fragment spanning the *sano* ORF was amplified and cloned into the pProEx expression vector (Life Technologies, Inc.). The construct was transformed into BL21-DE3 cells, from which Sano inclusion body preparations were made. Recombinant full-length protein was further purified from an SDS-polyacrylamide gel slice as described [90]. Rat polyclonal antibodies were generated by Covance, Inc. and used at a dilution of 1∶50.

### *sano* Mutant or Overexpression Clones in Pupal Wing

*sano* mutant or overexpressing clones were generated by the Flp-mediated recombination technique [91],[92]. Clones were induced either 36–48 or 48–60 hours AEL by a one hour heat shock (37°C). The genotype for *sano* mutant clones was *hs-FLP/+*; *ubi-GFP FRT^42^/sano^KO3^ FRT^42^*. The genotype for Sano flip-out clones was either *act>y^+^>Gal4/+*, *UAS-Sano-GFP/hs-FLP* or *act>y^+^>Gal4/+*, *arm-fz-GFP/+*; *UAS-Sano-HA/hs-FLP*.

### Phalloidin/Antibody Staining of Pupal Wings

Pupae were fixed 32–34 hours APF in 4% paraformaldehyde in PBS overnight (4°C). Pupal wings were dissected and washed several times in 0.5% PBST (0.5% Triton X-100 in PBS) and incubated with phalloidin-568 (Molecular Probes, 1∶1000) for one hour (ice). For antibody staining, pupae were fixed at 28 hours APF in 4% paraformaldehyde in PBS for one hour (4°C). Pupal wings were dissected and washed in 0.1% PBST. Wings were incubated in primary antibodies overnight (4°C) and then in secondary antibodies for two hours (ice).

### Eye Imaginal Disc Staining

*sano* mutant eye clones were generated using *eyeless*-FLP (*ey*-FLP). *sano* mutant cells were distinguished by the absence of the GFP signal. The 3^rd^ instar larvae were dissected in the PBS, fixed with fixation buffer (0.1M PIPES (pH6.9), 1mM EGTA (pH6.9), 1.0% Triton X-100, 2mM MgSO_4_, 1% formaldehyde), blocked in a solution (50mM Tris(pH6.8), 150mM NaCl, 0.1% Triton X-100, 5mg/ml bovine serum albumin (BSA)). The discs were incubated in primary antibodies in a washing/incubation solution (50mM Tris(pH6.8), 150mM NaCl, 0.1% Triton X-100, 1mg/ml BSA) overnight at 4°C and then in secondary antibodies for two hours at RT.

### Adult Eye Sections

*sevenless-Gal4* (*sev-Gal4*) was used to overexpress Sano in the eye. *sano* mutant eye clones were generated using *ey*-FLP. *sano* mutant cells were *w^+^/w^+^*, which can easily be distinguished from *w^+^/w^−^* heterozygous cells and from *w^−^/w^−^* twin spots in whole eyes. Since it is difficult to distinguish *w^+^*/*w^+^* versus *w^+^*/*w^−^* in thin sections, however, we chose only eyes with large mutant clones and adjacent *w^−^/w^−^* twin spots for sectioning. Fixation and semi-thin sectioning of the adult eyes were slightly modified from [93]. Sections from at least five independent eyes were analyzed for each genotype.

### Dorsal Trunk (DT) Length and Pupal Wing Cell Perimeter Measurements

Embryos were stained with 2A12 and projections from lateral views of confocal sections of the DT lumen of st. 16 embryos (at the four equal-compartment midgut stage) were traced from the starting point of metamere one to the point where the last transverse connective (TC) meets the DT in metamere nine using the Image J program (NIH). At least ten samples were measured and normalized to the length of the embryo for each genotype. An average length from three independent measurements of each sample was calculated.

Pupal wings were stained for E-Cadherin and the perimeter of pupal wing cells overexpressing Sano-GFP and of their wild-type neighbors were measured by Image J.

### Co-Immunoprecipitation and Western Blotting

*da-Gal4/UAS-Sano-HA*; *dsh-GFP/+* embryos were used for co-IP, and *da-Gal4/UAS-Sano-HA* and *dsh-GFP* embryos were used as controls. The embryos were collected and homogenized in radioimmunoprecipitation (RIPA) buffer (Cell Signaling) including protease inhibitor cocktail (Roche). A small aliquot of the cleared supernatant was used for the Western to check the protein input with α-GFP and α-HA. Dynabeads Protein G (Invitrogen) was incubated with mouse α-HA (Roche) or rabbit α-GFP (Molecular Probes) for 10 minutes at RT. After several washes with PBTw (0.01% Tween-20 in 1× PBS), the remaining supernatant was incubated with antibody-bound Dynabeads Protein G for 20 minutes at RT. The beads were washed three times with RIPA buffer, and boiled in SDS sample buffer to elute the proteins. Bound antigen was detected by enhanced chemiluminescence (GE Healthcare). The antibodies for Western blotting were used at the following concentrations: rat α-HA (Roche, 1∶2,000), mouse α-GFP (Roche, 1∶2,000). Co-Ips were repeated three times with the same results.

### Northern Hybridization

The developmental Northern blot was prepared as described [94] and hybridized with a *Bgl* II/*Not* I fragment from pPB3 cDNA labeled by random priming.

### Dextran Injections

Fluorescence-labelled 10kDa dextran (Molecular Probes) injections were performed as described [22],[95], using wild-type embryos as a negative control and the *mega* mutant as a positive control.

### ApopTag Staining

Embryos were dechorinated and fixed in the fixative for 20–30 min at RT. The fixative includes 800µl 5× buffer B (50mM KPO_4_, pH6.8, 225mM KCl, 75mM NaCl, 65mM MgCl_2_), 800ml 37% formaldehyde, 2.5ml dH_2_O and 8ml heptane. Antibody staining was performed with α-Tgo antibody to mark the tracheal nuclei. After secondary antibody labelling, the embryos were treated with 10µg/ml proteinase K for 1 min and post-fixed with 3.7% formaldehyde in 0.1% Tween20 in 1× PBS (PBT). ApopTag staining was performed using ApopTag Plus Peroxidase *In Situ* Apoptosis Kit (Millipore, S7101), and the cells undergoing apoptosis were labeled with rhodamine-conjugated α-Dig antibody (1∶10, Roche).

## Supporting Information

Figure S1 Developmental Northern of *sano*. (A) *sano* transcript is most abundant in 8–12 hour embryos and is not maternally contributed (note absence of expression in 0–2 hour embryos). *sano* is not expressed in *Drosophila* S2 cells (last lane of gel, S).
(0.62 MB TIF)

Figure S2 *sano* expression in *trhH99* mutants. (A–F) *sano* mRNA expression in st.10–12 wild-type (A, C, E) and *trh^1^H99* mutant (B, D, F) embryos. Weak tracheal expression is observed in *trhH99* mutants at early stages (arrows in B, D, F). (G and H) *sano* expression in st.13 wild-type (G) and *trh^1^H99* mutant (H) embryos. *sano* mRNA is completely absent in the trachea (arrows in G and H), whereas the midline expression is still strong in the *trhH99* mutant (arrowheads in G and H).
(3.82 MB TIF)

Figure S3 Generation of *sano* null alleles by homologous recombination. (A) Schematic diagram for *sano* knockout and the primers used for diagnostic PCRs. (B) Genomic PCRs for the three knockout mutants KO1, KO2 and KO3. The original transgenic fly line carrying the knockout transgenic construct is used as a negative control. (C–H) *sano* mRNA expression in wild-type embryos (C, E, G) and in embyos transheterozygous for a *sano* null mutant over a deficiency that removes sano (D, F, H). *sano* expression is absent in *sano^KO1^/Df(2R)Exel6088* (D), *sano^KO2^/Df(2R) Exel6088* (F), and *sano^KO3^/Df(2R)Exel6088* (H) embryos.
(2.79 MB TIF)

Figure S4 Known pathways affecting tracheal tube length are unaffected in sano mutant embryos. (A–D) Chitin cable and a chitin-modifying enzyme show normal levels and distribution in sano mutants. Chitin-binding protein (CBP; A, B). α-Vermiform (Verm; C, D). (E and F) Septate junction marker α-Coracle (Cor) shows normal distribution. (G–I) Barrier function is intact in *sano* null trachea. Dye exclusion assay in wild-type, *sano* and *mega* embryos. The fluorescent-conjugated 10 kDa dextran did not diffuse into the tracheal lumen in wild-type and *sano^KO2^* mutants (G and H), whereas it rapidly crosses the tracheal epithelium to fill the luminal space in *mega^EA97^* mutants (I). All embryos shown are st.16. Scale bars: 20 µm in (A, B) and (G–I). 10 µm in (C–F).
(2.43 MB TIF)

Figure S5 No significant increase of apoptosis is detected in Sano-overexpressing trachea. (A, B) Compared to WT (A), no significant increase of apoptosis was detected in the Sano-overexpressing tracheal cells even when a huge gap was seen (B). The images are metameres 2–4 of the st.16 embryos. Green, Tgo; red, Apoptag. Scale bars: 20 µm.
(5.13 MB TIF)

Figure S6 *sano* expression in the imaginal discs. (A–C) β-gal expression of *rp395* in the imaginal discs of 3rd instar larvae. (D–F) *sano* mRNA expression in wild-type imaginal discs.
(3.71 MB TIF)

Figure S7 Examples of Sano-overexpressing clones in the pupal wings. (A) Cartoon image indicates regions where Sano overexpression causes non-autonomous polarity defects in adjacent wild-type cells. (B) Sano overexpression sometimes causes multiple wing hairs (arrows), another typical PCP phenotype. (C and D) Examples of nonautonomous PCP defects in Sano-overexpressing clones. (C) Unlike the other genes showing nonautonomy, nonautonomous effects caused by Sano overexpression have no directionality. Some WT hairs near the clones point toward the clones (arrows), whereas others point away from the clones (arrowheads). (D) Hair formation delay is shown inside the clones (asterisks), whereas the polarity defects are observed outside of the clones of Sano overexpressing cells (arrows). Scale bars: 5 µm in (B), 10 µm in (C, D).
(3.60 MB TIF)

Figure S8 Sano misexpression disrupts the asymmetric distribution of all PCP proteins analyzed. (A) Stbm (red) loses asymmetric localization in Sano-overexpressing region (green). The adjacent wild-type cells in B look smaller because they are in the vein (brackets). Scale bars: 5 µm. (B) Dsh (red) is distributed throughout the apical margin in Sano-overexpressing clones (green).
(2.83 MB TIF)

Figure S9 *sano* LOF clones in the wings. (A, B) Examples of *sano* loss-of-function (LOF) clones (absence of GFP) showing normal hair cell polarity in the pupal wings. (C, D) Some *sano* clones induced early show only twin spots (bright GFP signal, arrows). Scale bars: 10 µm in (A, B); 50 µm in (C, D).
(3.26 MB TIF)

Figure S10 Reduction of dsh dosage suppresses gain-of-function phenotype of Sano. (A, B) Examples of *MS1096-Gal4/+*; *UAS-Sano/+* thorax. (C, D) Examples of *MS1096-Gal4/dsh^1^*; *UAS-Sano/+* thorax. Reducing the PCP function of *dsh* suppresses Sano-overexpressing PCP phenotypes. All flies shown are female and the crosses were done at 18°C.
(4.14 MB TIF)

Figure S11 Dsh and Fmi localization in the WT trachea. (A, B) Dsh localization in the WT tracheal cells. At early stages, Dsh localizes in the cytoplasm (A), and at later stages, it shows enrichment at the apical membrane (B). Green, Dsh; Red, SAS. (C–E) Fmi localization in the WT tracheal cells. During tracheal morphogenesis, Fmi is detected at the adherens junction in the trachea showing colocalization with E-Cad. Red, Fmi; Blue, E-Cad; Green, SAS.
(4.97 MB TIF)
