# Supplementary figures and images for: Serrano (Sano) Functions with the Planar Cell Polarity Genes to Control Tracheal Tube Length
Source: PLoS Genet. 2009 Nov 26;5(11):e1000746. doi: 10.1371/journal.pgen.1000746 (PMC2776533; doi:10.1371/journal.pgen.1000746)

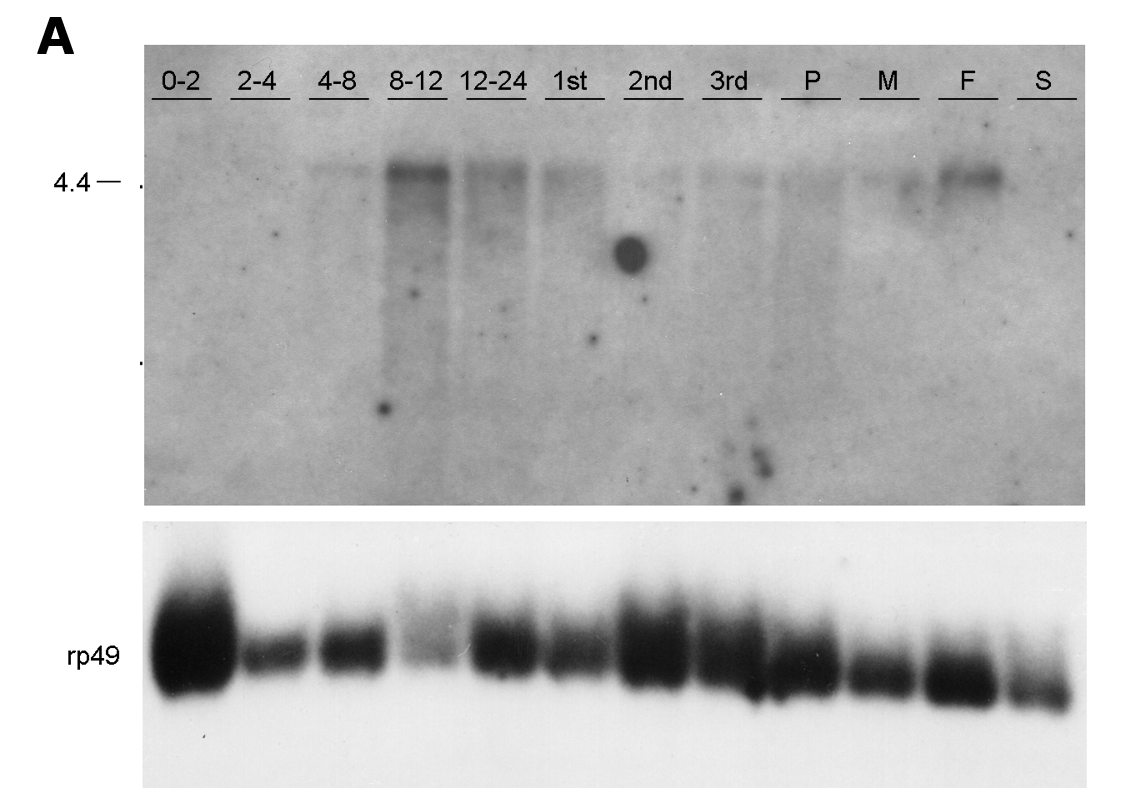

Supplement: Figure S1 — Developmental Northern of sano. (A) sano transcript is most abundant in 8–12 hour embryos and is not maternally contributed (note absence of expression in 0–2 hour embryos). sano is not expressed in Drosophila S2 cells (last lane of gel, S). (0.62 MB TIF) [file pgen.1000746.s001.tif]

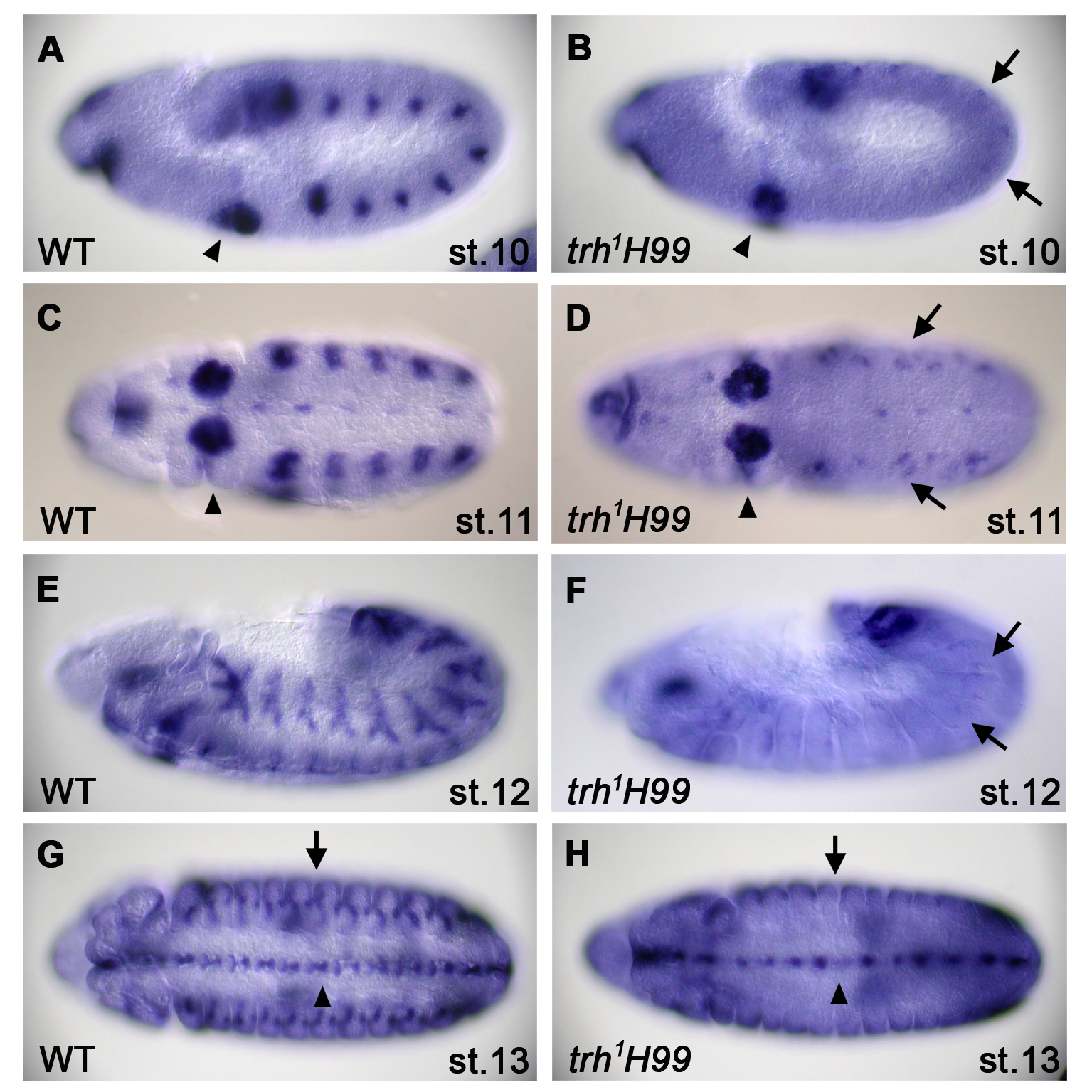

Supplement: Figure S2 — sano expression in trhH99 mutants. (A–F) sano mRNA expression in st.10–12 wild-type (A, C, E) and trh1H99 mutant (B, D, F) embryos. Weak tracheal expression is observed in trhH99 mutants at early stages (arrows in B, D, F). (G and H) sano expression in st.13 wild-type (G) and trh1H99 mutant (H) embryos. sano mRNA is completely absent in the trachea (arrows in G and H), whereas the midline expression is still strong in the trhH99 mutant (arrowheads in G and H). (3.82 MB TIF) [file pgen.1000746.s002.tif]

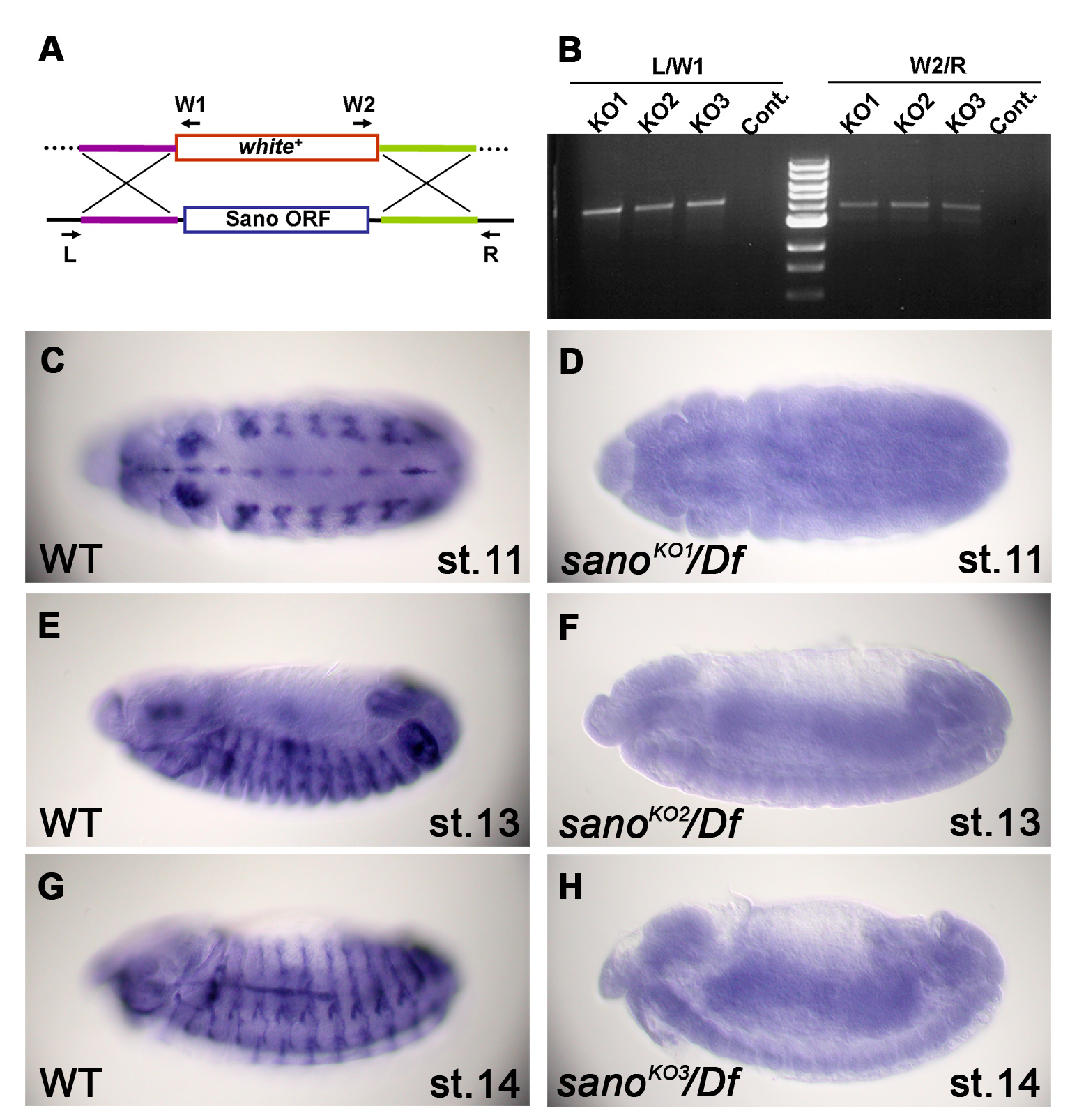

Supplement: Figure S3 — Generation of sano null alleles by homologous recombination. (A) Schematic diagram for sano knockout and the primers used for diagnostic PCRs. (B) Genomic PCRs for the three knockout mutants KO1, KO2 and KO3. The original transgenic fly line carrying the knockout transgenic construct is used as a negative control. (C–H) sano mRNA expression in wild-type embryos (C, E, G) and in embyos transheterozygous for a sano null mutant over a deficiency that removes sano (D, F, H). sano expression is absent in sanoKO1/Df(2R)Exel6088 (D), sanoKO2/Df(2R) Exel6088 (F), and sanoKO3/Df(2R)Exel6088 (H) embryos. (2.79 MB TIF) [file pgen.1000746.s003.tif]

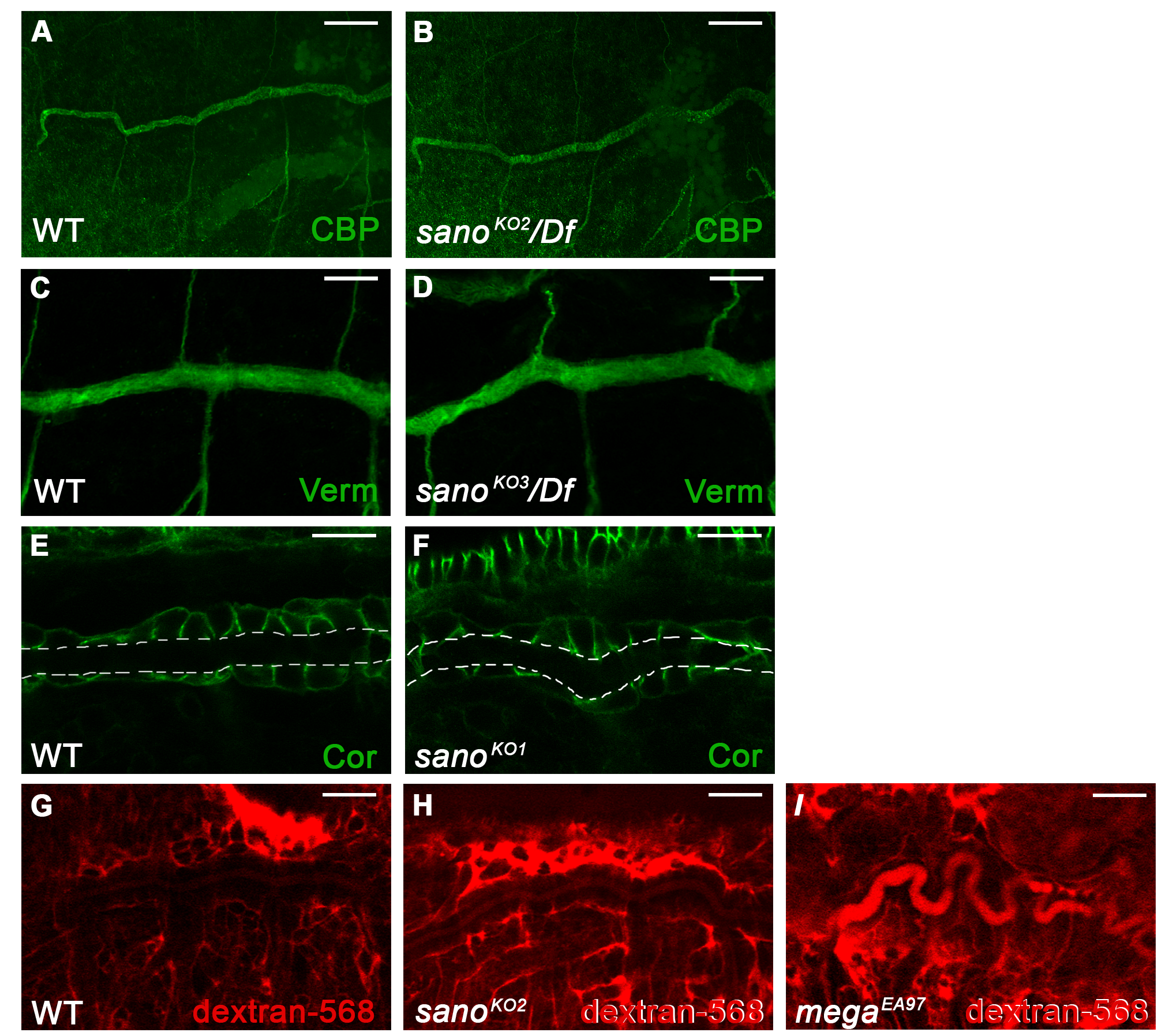

Supplement: Figure S4 — Known pathways affecting tracheal tube length are unaffected in sano mutant embryos. (A–D) Chitin cable and a chitin-modifying enzyme show normal levels and distribution in sano mutants. Chitin-binding protein (CBP; A, B). α-Vermiform (Verm; C, D). (E and F) Septate junction marker α-Coracle (Cor) shows normal distribution. (G–I) Barrier function is intact in sano null trachea. Dye exclusion assay in wild-type, sano and mega embryos. The fluorescent-conjugated 10 kDa dextran did not diffuse into the tracheal lumen in wild-type and sanoKO2 mutants (G and H), whereas it rapidly crosses the tracheal epithelium to fill the luminal space in megaEA97 mutants (I). All embryos shown are st.16. Scale bars: 20 µm in (A, B) and (G–I). 10 µm in (C–F). (2.43 MB TIF) [file pgen.1000746.s004.tif]

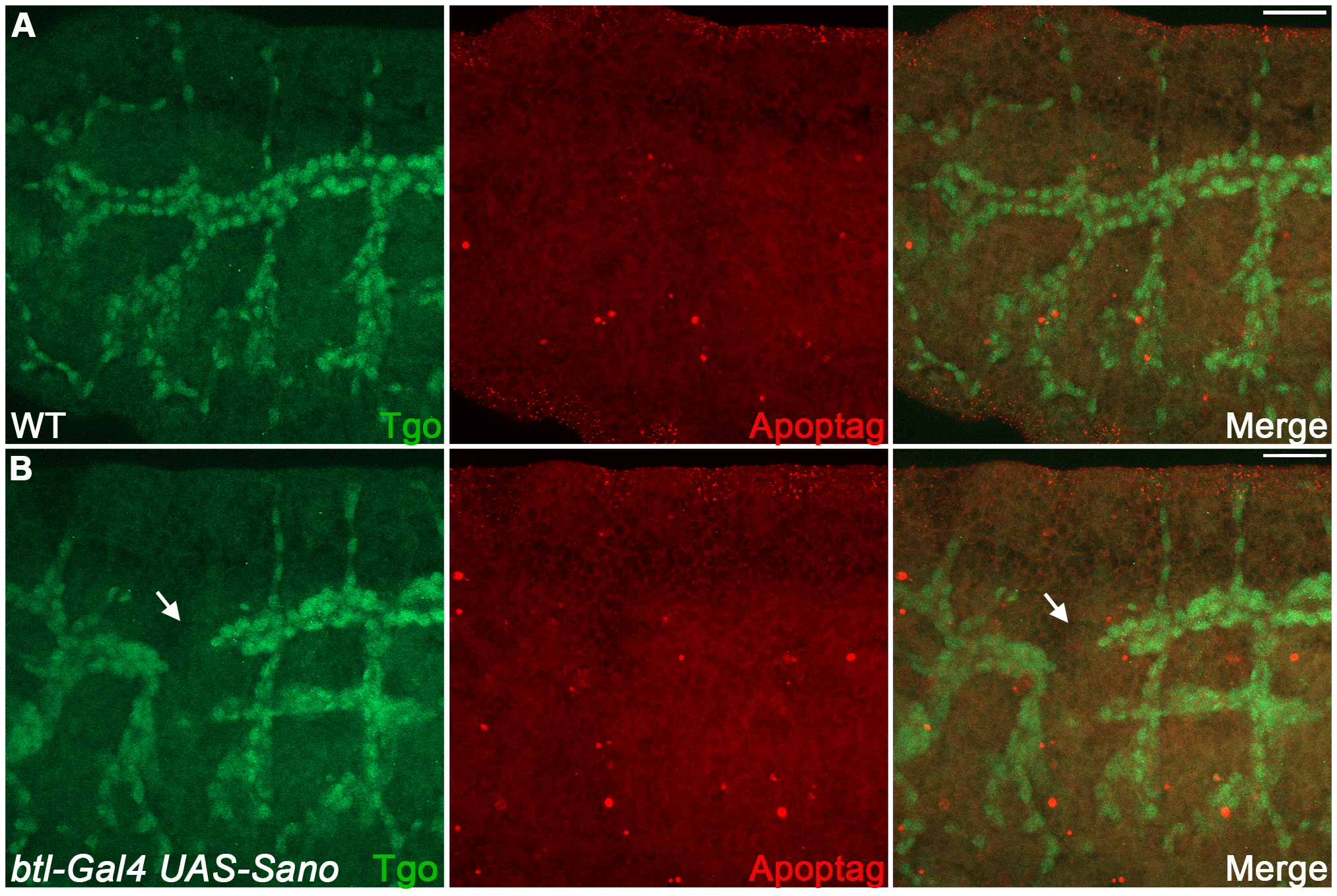

Supplement: Figure S5 — No significant increase of apoptosis is detected in Sano-overexpressing trachea. (A, B) Compared to WT (A), no significant increase of apoptosis was detected in the Sano-overexpressing tracheal cells even when a huge gap was seen (B). The images are metameres 2–4 of the st.16 embryos. Green, Tgo; red, Apoptag. Scale bars: 20 µm. (5.13 MB TIF) [file pgen.1000746.s005.tif]

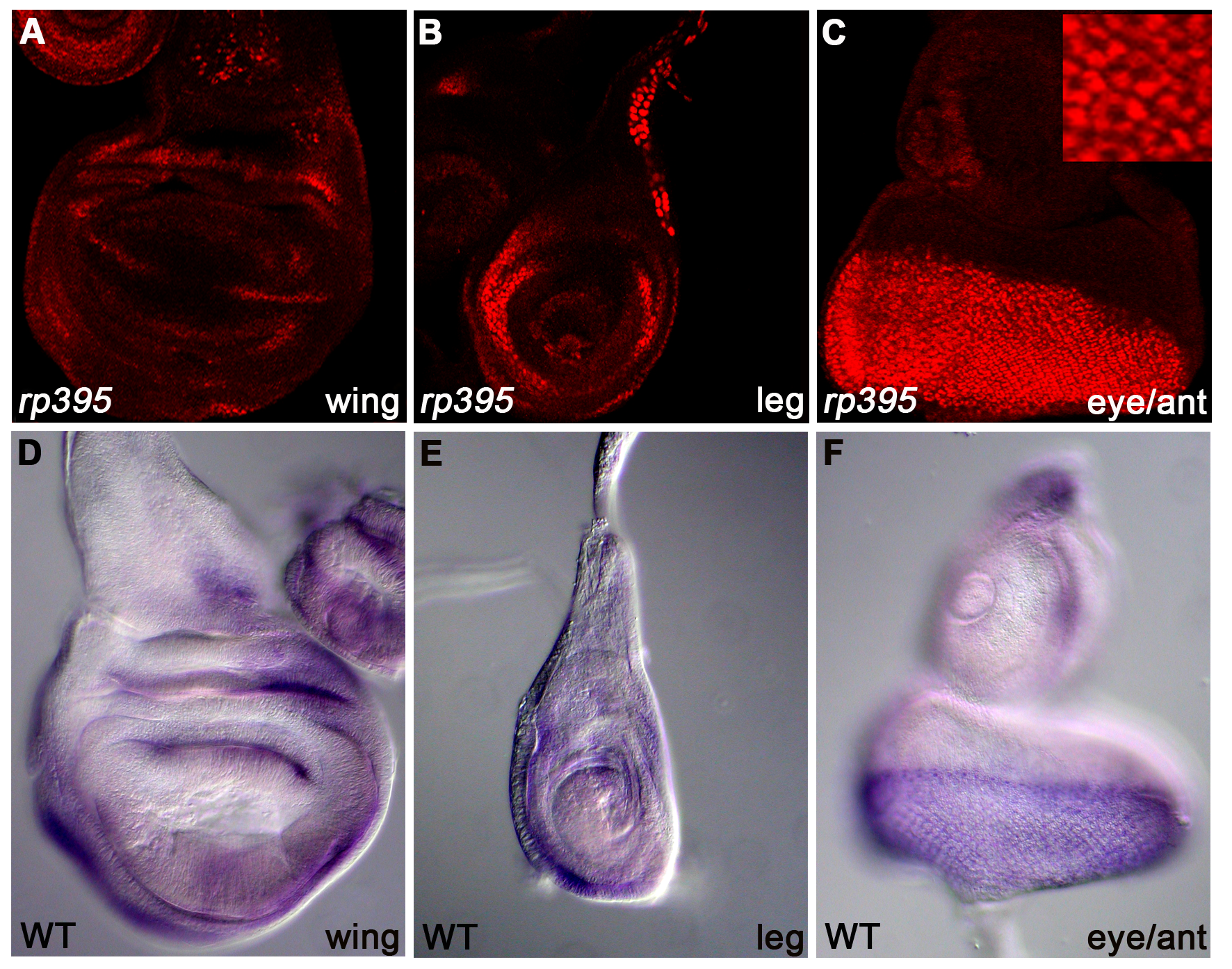

Supplement: Figure S6 — sano expression in the imaginal discs. (A–C) β-gal expression of rp395 in the imaginal discs of 3rd instar larvae. (D–F) sano mRNA expression in wild-type imaginal discs. (3.71 MB TIF) [file pgen.1000746.s006.tif]

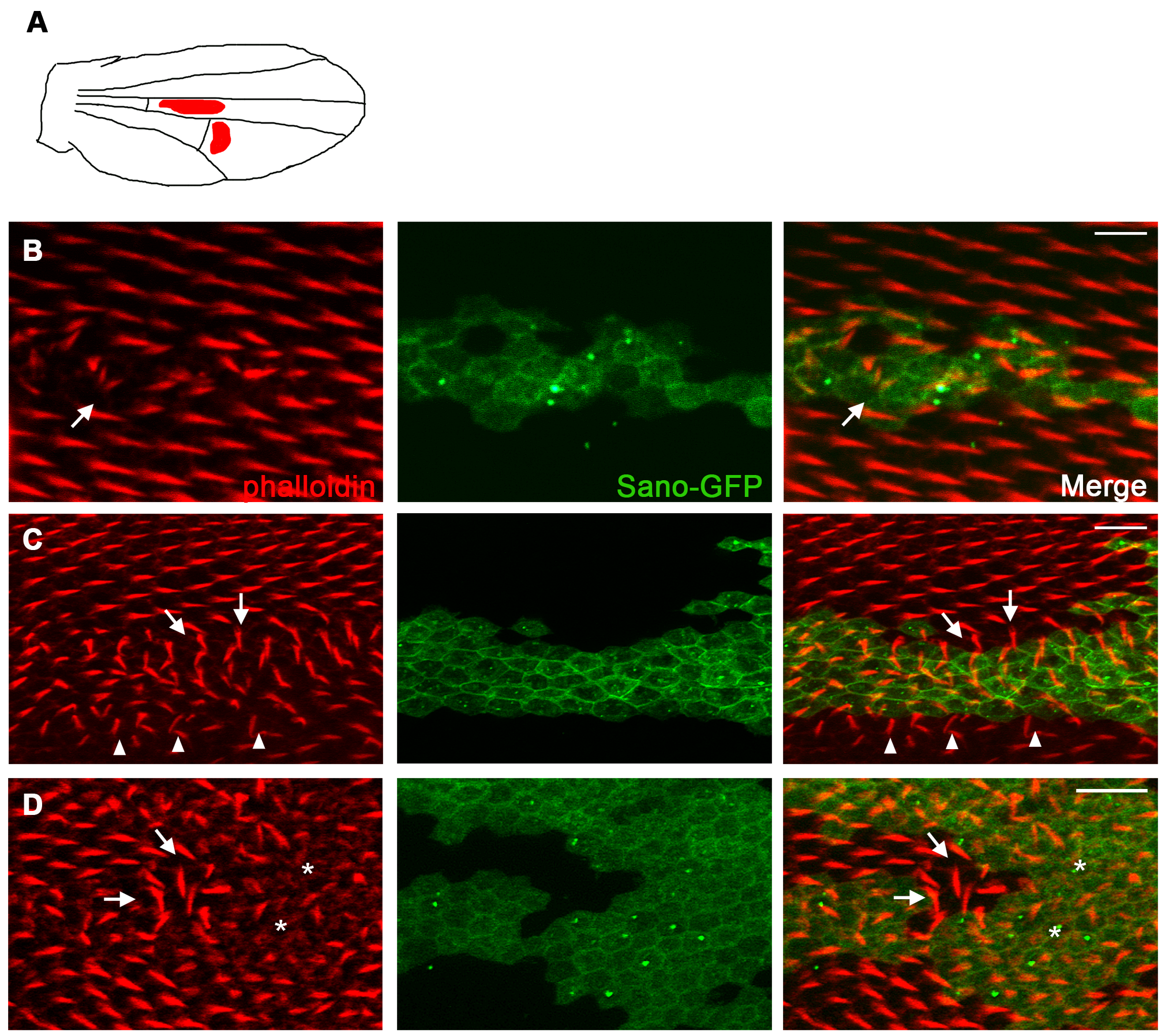

Supplement: Figure S7 — Examples of Sano-overexpressing clones in the pupal wings. (A) Cartoon image indicates regions where Sano overexpression causes non-autonomous polarity defects in adjacent wild-type cells. (B) Sano overexpression sometimes causes multiple wing hairs (arrows), another typical PCP phenotype. (C and D) Examples of nonautonomous PCP defects in Sano-overexpressing clones. (C) Unlike the other genes showing nonautonomy, nonautonomous effects caused by Sano overexpression have no directionality. Some WT hairs near the clones point toward the clones (arrows), whereas others point away from the clones (arrowheads). (D) Hair formation delay is shown inside the clones (asterisks), whereas the polarity defects are observed outside of the clones of Sano overexpressing cells (arrows). Scale bars: 5 µm in (B), 10 µm in (C, D). (3.60 MB TIF) [file pgen.1000746.s007.tif]

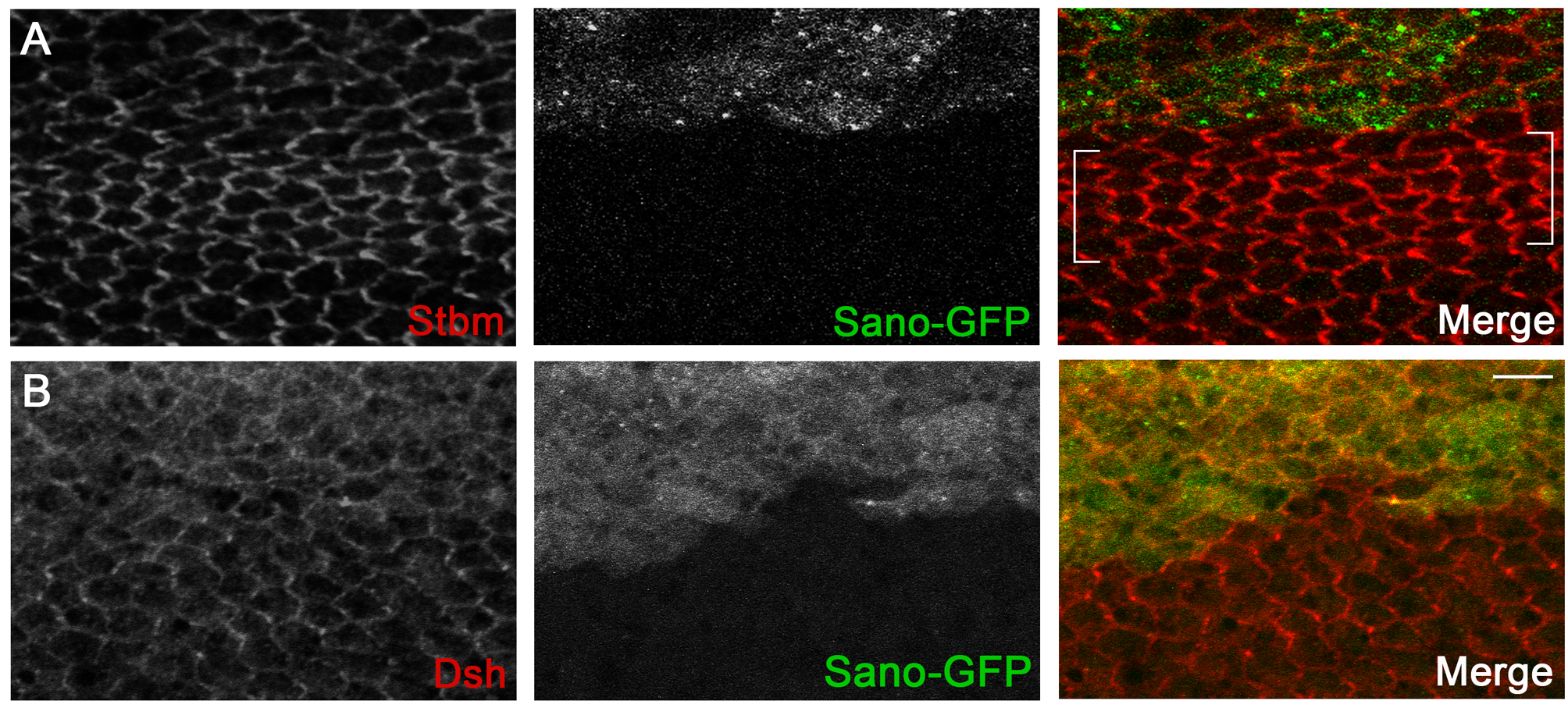

Supplement: Figure S8 — Sano misexpression disrupts the asymmetric distribution of all PCP proteins analyzed. (A) Stbm (red) loses asymmetric localization in Sano-overexpressing region (green). The adjacent wild-type cells in B look smaller because they are in the vein (brackets). Scale bars: 5 µm. (B) Dsh (red) is distributed throughout the apical margin in Sano-overexpressing clones (green). (2.83 MB TIF) [file pgen.1000746.s008.tif]

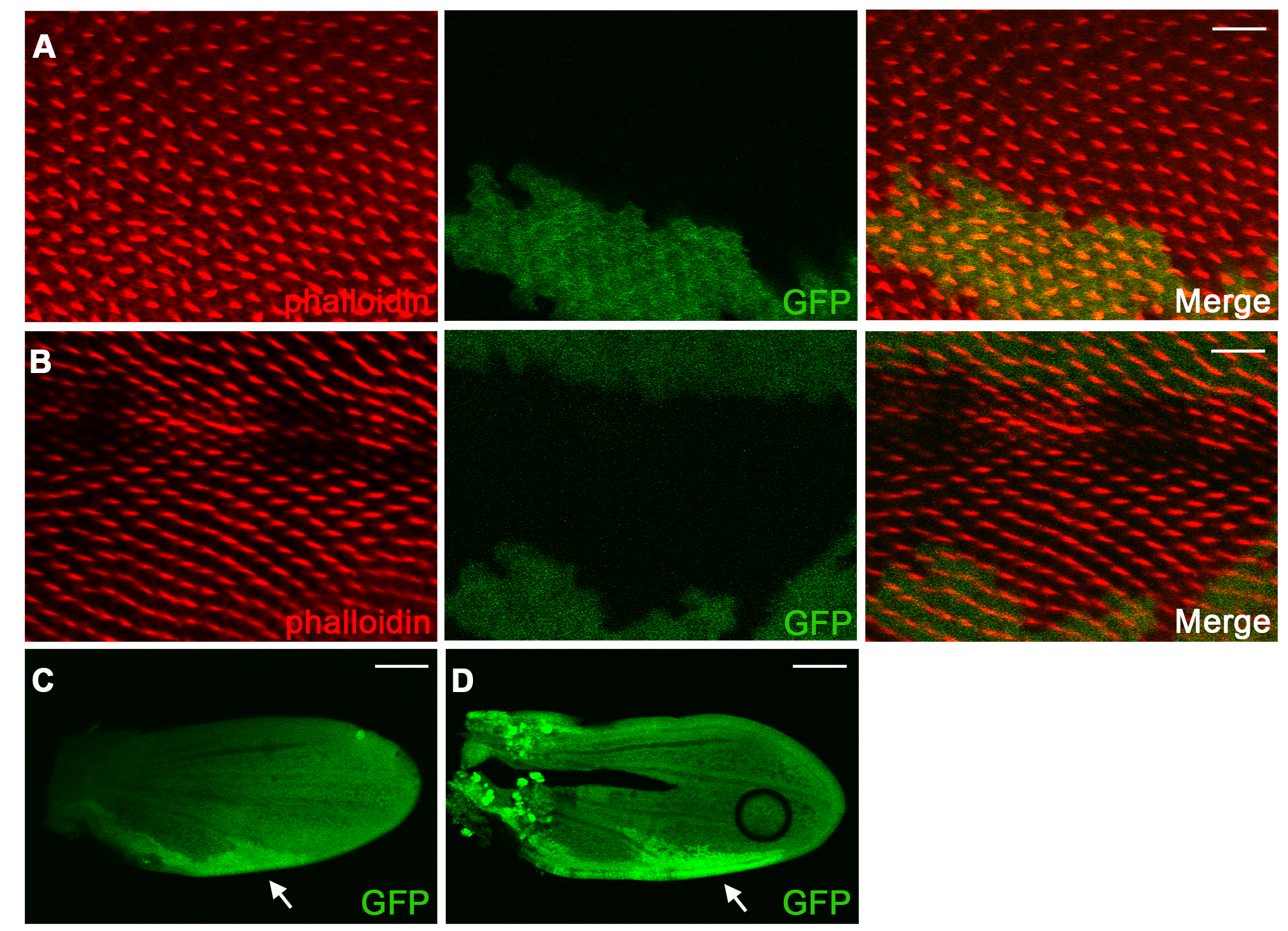

Supplement: Figure S9 — sano LOF clones in the wings. (A, B) Examples of sano loss-of-function (LOF) clones (absence of GFP) showing normal hair cell polarity in the pupal wings. (C, D) Some sano clones induced early show only twin spots (bright GFP signal, arrows). Scale bars: 10 µm in (A, B); 50 µm in (C, D). (3.26 MB TIF) [file pgen.1000746.s009.tif]

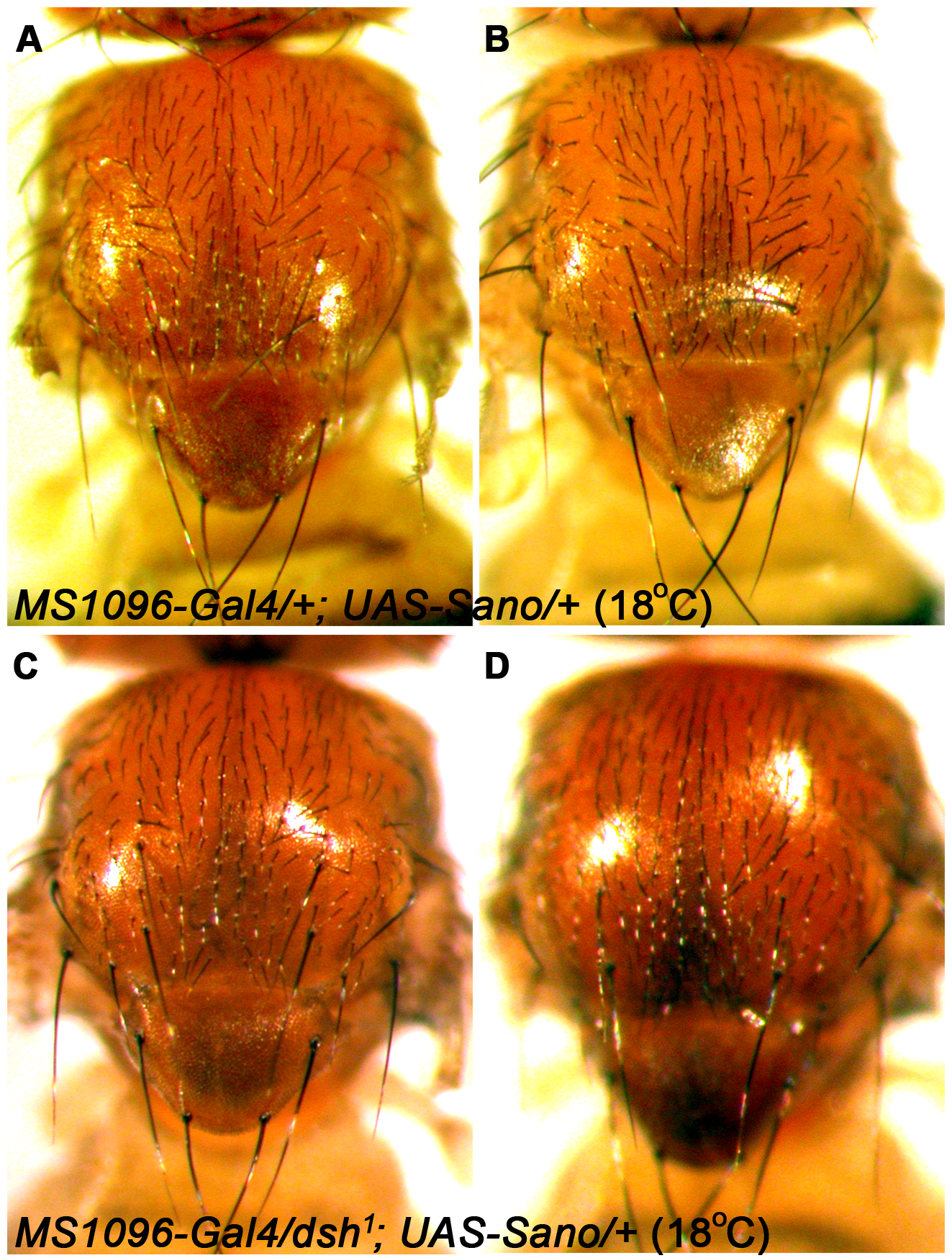

Supplement: Figure S10 — Reduction of dsh dosage suppresses gain-of-function phenotype of Sano. (A, B) Examples of MS1096-Gal4/+; UAS-Sano/+ thorax. (C, D) Examples of MS1096-Gal4/dsh1; UAS-Sano/+ thorax. Reducing the PCP function of dsh suppresses Sano-overexpressing PCP phenotypes. All flies shown are female and the crosses were done at 18°C. (4.14 MB TIF) [file pgen.1000746.s010.tif]

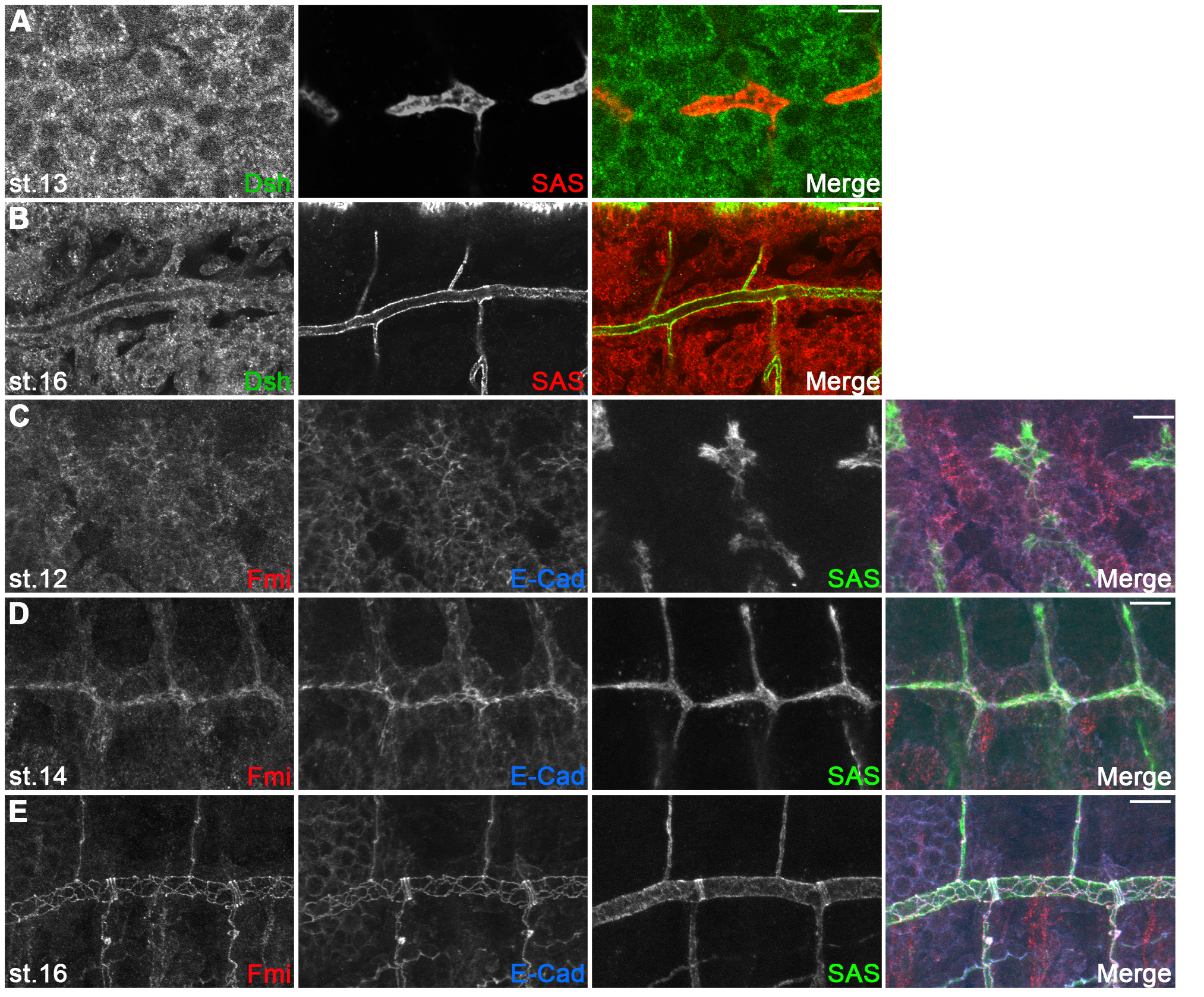

Supplement: Figure S11 — Dsh and Fmi localization in the WT trachea. (A, B) Dsh localization in the WT tracheal cells. At early stages, Dsh localizes in the cytoplasm (A), and at later stages, it shows enrichment at the apical membrane (B). Green, Dsh; Red, SAS. (C–E) Fmi localization in the WT tracheal cells. During tracheal morphogenesis, Fmi is detected at the adherens junction in the trachea showing colocalization with E-Cad. Red, Fmi; Blue, E-Cad; Green, SAS. (4.97 MB TIF) [file pgen.1000746.s011.tif]
